# Elevating seed oil content in a polyploid crop by induced mutations in *SEED FATTY ACID REDUCER* genes

**DOI:** 10.1111/pbi.13381

**Published:** 2020-04-13

**Authors:** Nirosha L. Karunarathna, Haoyi Wang, Hans‐Joachim Harloff, Lixi Jiang, Christian Jung

**Affiliations:** ^1^ Plant Breeding Institute Christian‐Albrechts‐University of Kiel Kiel Germany; ^2^ Institute of Crop Science Zhejiang University Hangzhou China

**Keywords:** rapeseed, *Brassica napus*, CRISPR‐Cas, EMS, genome editing, mutagenesis, GDSL, *SFAR*

## Abstract

Plant‐based oils are valuable agricultural products, and seed oil content (SOC) is the major yield component in oil crops. Increasing SOC has been successfully targeted through the selection and genetic modification of oil biosynthesis. The SOC in rapeseed declined during the seed maturation and eventually caused the final accumulated seed oil quantity. However, genes involved in oil degradation during seed maturity are not deeply studied so far. We performed a candidate gene association study using a worldwide collection of rapeseed germplasm. We identified *SEED FATTY ACID REDUCER* (*SFAR*) genes, which had a significant effect on SOC and fatty acid (FA) composition. *SFAR* genes belong to the GDSL lipases, and GDSL lipases have a broad range of functions in plants. After quantification of gene expression using RNA‐seq and quantitative PCR, we used targeted (CRISPR‐Cas mediated) and random (chemical) mutagenesis to modify turnover rates of seed oil in winter rapeseed. For the first time, we demonstrate significant increase of SOC in a crop after knocking out members of the *BnSFAR4* and *BnSFAR5* gene families without pleiotropic effects on seed germination, vigour and oil mobilization. Our results offer new perspectives for improving oil yield by targeted mutagenesis.

## Introduction

In the seeds of oil plants, triacylglycerols (TAGs) are the major energy resource required during germination. Seed oil content (SOC) and fatty acid (FA) composition are major determinants of yield and quality. The *de novo* synthesis of FAs occurs in plastids. Then, TAGs are further assembled in the endoplasmic reticulum (Li‐Beisson *et al.*, 2013, Graham, 2008) and, surrounded by a lipid monolayer and oil body proteins, so‐called oil bodies are deposited in the cytosol (Li‐Beisson *et al.*, 2013; Xu and Shanklin, 2016). TAGs can be degraded into free FAs by various lipases (Li‐Beisson *et al.*, 2013). The free FAs are subjected to β‐oxidation, a catabolic process by which FA molecules are broken down to produce acetyl‐CoA, which is subsequently converted into 4‐carbon compounds via the glyoxylate cycle (Pracharoenwattana and Smith, 2008, Borek et al., 2015, Graham, 2008). Previous studies reported that SOC peaks at seed maturation (Kelly *et al.*, 2013, Wan et al., 2017) and then degrades during seed desiccation indicating that the accumulation of lipids in seeds is not simply a unilateral synthesis process, but a dynamic balance between anabolism and catabolism, influenced by numerous external and internal factors (Kurat *et al.*, 2006; Zhou *et al.*, 2018). Our knowledge about lipid decomposition during seed maturation is comparatively limited (Ding *et al.*, 2019; Kanai *et al.*, 2019; Kelly *et al.*, 2013), and the biological implication of TAG degradation during seed development remains to be elucidated.

GDSL lipases play an important role in TAG degradation. Their name comes from the highly conserved GDSL amino acid motif near the N‐terminus. GDSL lipases contain five consensus domain blocks (I‐V) forming the catalytical active serine–aspartate–histidine triad with the oxyanion hole residues serine (S), glycine (G) and asparagine (N) (Akoh *et al.*, 2004; Upton and Buckley, 1995). GDSL lipases were identified in many species, including microbes, animals and plants (Akoh *et al.*, 2004; Arif *et al.*, 2004; Brick *et al.*, 1995; Cummins and Edwards, 2004; Horne *et al.*, 2009; Pringle and Dickstein, 2004). The advancement of genome sequencing projects revealed more than 1100 members of GDSL lipases from the model plant *Arabidopsis thaliana*, green algae, moss, fern, grapevine, tree and crop species (Chepyshko *et al.*, 2012; Volokita *et al.*, 2011)*.* The GDSL family from *Arabidopsis* consists of 105 members (Lai *et al.*, 2017). *GDSL* genes have flexible active sites (Akoh *et al.*, 2004; Shakiba *et al.*, 2016), which change conformation after binding of different substrates. In plants, *GDSL* genes play roles in the regulation of morphological development, pathogen defence, abiotic stress and secondary metabolism (An *et al.*, 2019; Kim *et al.*, 2013; Lai *et al.*, 2017). However, the functions of most of the GDSL enzymes are little understood.

Rapeseed (*Brassica napus*) is the only important oil crop of northern latitudes. The SOC is ranging between 26 and 50% (Xiao *et al.*, 2019). Rapeseed and its closely related oilseed crops *B. juncea* and *B. rapa* belong to the Cruciferae plant family together with *Arabidopsis.* Its allopolyploid genome resulted from a spontaneous hybridization between *B. rapa* and *B. oleracea* (AACC, 2n = 38) which took place ca. 7500 years ago (Chalhoub *et al.*, 2014). As a consequence, one gene in *Arabidopsis* corresponds to multiple orthologous copies in the rapeseed genome. Therefore, duplicated genes may undergo neo/subfunctionalization (Conant and Wolfe, 2008; Xia *et al.*, 2016) and translating knowledge directly from *Arabidopsis* is relatively difficult. The availability of the reference genome ‘Darmor*‐bzh*’ facilitates the identification of *Arabidopsis* orthologs in rapeseed and the exploration of their biological function (Chalhoub *et al.*, 2014).

Mutations are instrumental in understanding gene functions. Traditionally, random mutations induced by chemicals or irradiation were mainly used for functional analysis. However, random mutagenesis has several limitations when it comes to practical breeding, especially in polyploid crops like rapeseed, where several genes have to be mutagenized simultaneously to gain the desired phenotype (Jung *et al.*, 2018). During the last decades, targeted genome editing techniques evolved rapidly as a more efficient alternative to classical approaches. In the case of rapeseed, the CRISPR‐Cas technology proved to be a powerful tool (Doudna and Charpentier, 2014) to create multiple mutations within gene families (Braatz *et al.*, 2017).

This study aimed to elevate the SOC in rapeseed. In the past, numerous studies investigated the increase in SOC by overexpression of genes critical for the lipid biosynthesis pathway (Elahi *et al.*, 2016; Vigeolas *et al.*, 2007; Zafar *et al.*, 2019). So far, little effort was put in preventing the synthesized lipids from decomposing during seed maturation (Ding *et al.*, 2019; Kanai *et al.*, 2019; Kelly *et al.*, 2013). Here, we identified *GDSL* genes in the rapeseed genome for the first time and studied their expression in developing seeds. With a candidate gene association study using a worldwide collection of rapeseed accessions, we identified *SEED FATTY ACID REDUCER* (*SFAR*) genes, which had a significant effect on SOC. We used both chemically induced mutagenesis and CRISPR‐Cas‐mediated gene editing for the functional characterization of *BnSFAR* genes. We demonstrate that only the simultaneous knockout of several *BnSFAR4* and *BnSFAR5* genes increases the SOC without adverse effect on seed germination and vigour, thus providing a successful example to increase rapeseed SOC by pyramiding *BnSFAR* knockout alleles. Furthermore, we demonstrate the superiority of CRISPR‐Cas technology over random mutagenesis if the aim is to knockout multiple gene copies in polyploid species.

## Results

### Identification of *GDSL* genes in the rapeseed genome

**Figure 1:**
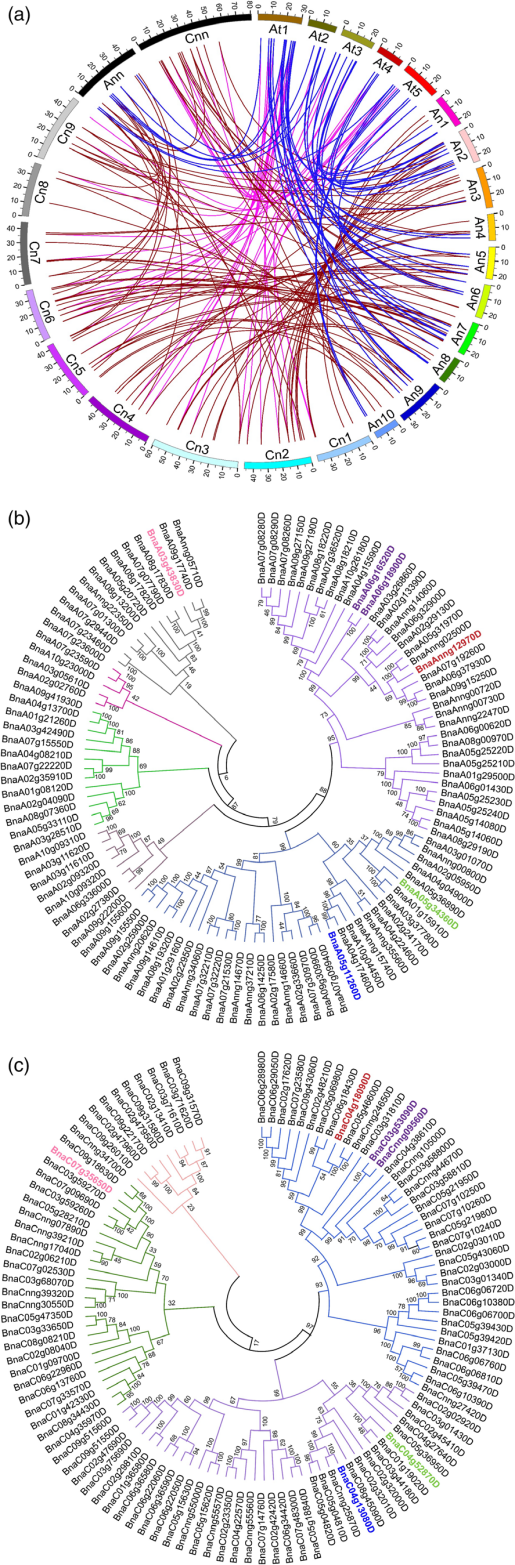
Position of *GDSL* genes in the genomes of *A. thaliana* and *B. napus* and synteny relationships between the paralogous and/or orthologous genes and unrooted phylogenetic trees (a) Chromosomes of *A. thaliana* are named as At1 through At5. Chromosomes of the A and C subgenomes of rapeseed are named as Anx and Cnx, respectively. ‘Ann’ and ‘Cnn’ represent the unmapped sequence reads. The blue lines are connecting the orthologs between *Arabidopsis* and the A subgenome of rapeseed. The pink lines are connecting the orthologs between Arabidopsis and the C subgenome of rapeseed. The brown lines are connecting the paralogs between the A and C subgenomes of rapeseed. Phylogenetic tree of 222 *BnGDSL* genes from the A (b) and C (c) subgenomes of rapeseed. *BnGDSL* genes are grouped into six clusters, indicated by different colours. The amino acid sequences were aligned using ClustalW2 (default parameters), and the phylogenetic trees were constructed using the neighbour‐joining method. *BnSFAR1‐BnSFAR5* genes are shown in red, blue, green, purple and pink, respectively.

We performed BLAST using 105 annotated *GDSL* genes from *Arabidopsis* (http://www.arabidopsis.org/) against the rapeseed reference genome. We found 222 genes with *GDSL* domains equally distributed between the A and C subgenomes of rapeseed (Figure S1). Chromosome A07 has the highest abundance of *BnGDSL* genes (17 out of 222) (Figure S1a). We applied multi‐to‐multi, multi‐to‐one and one‐to‐one models for sequence alignment. Each *AtGDSL* gene had one or more orthologs in the rapeseed genome. *GDSL* gene positions across the two genomes and the syntenic relationship between the paralogs and/or orthologs are shown in Figure 1a. Rapeseed orthologs of *AtGDSL* genes showed high inter‐ and intra‐species sequence similarities confirming a short evolutionary distance between rapeseed and *Arabidopsis*. To further study the relationships between *GDSL* genes from the A and C subgenomes, we performed a phylogenetic analysis using the neighbour‐joining method (Figure 1b,c). According to the degree of sequence similarity, 111 *BnGDSLs* each from the A and C subgenomes were assigned to 6 and 4 clades, respectively. The cluster analysis of the two subgenomes also uncovered substantial sequence variations between *BnGDSL* genes. The overall sequence diversity of *BnGSDLs* within the C subgenome (Figure 1c) is lower than in the A subgenome (Figure 1b).

We first considered five known *SFAR* genes that function to decrease SOC in *Arabidopsis* and searched for rapeseed homologs (Chen *et al.*, 2012). BLAST analysis using AtSFAR1‐AtSFAR5 protein sequences as queries for the rapeseed database resulted in many hits. We considered different parameters: e‐value (0), gene identity (>80%) and the presence of conserved domains to select the putative rapeseed orthologs. Subsequently, 12 homologous genes were identified, two paralogs in each gene family, except for *BnSFAR4* where four paralogs have been found (Table S1). We further divided *BnSFAR4* into subfamily‐a and subfamily‐b based on their sequence similarity. All the conserved blocks were found in all candidate genes.

### Sequence variations within *BnSFAR* genes impacting seed oil content

**Figure 2:**
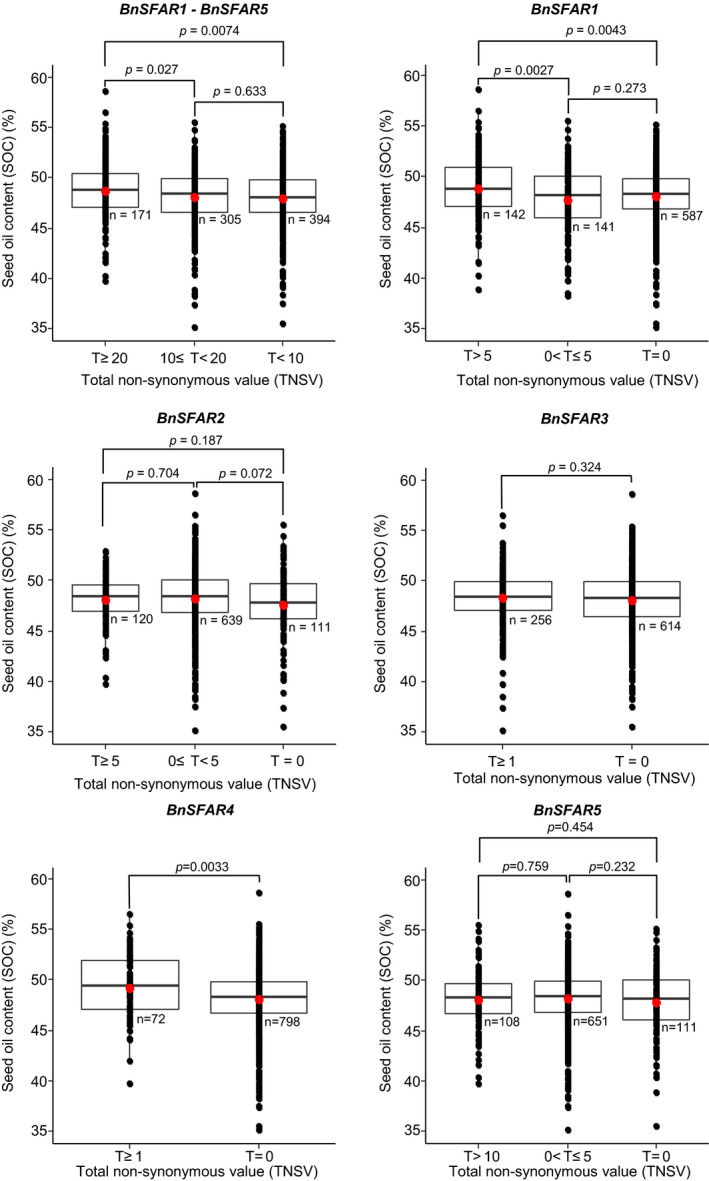
The effect of non‐synonymous single nucleotide polymorphisms (SNP) within *BnSFAR* genes on seed oil content in 870 non‐related rapeseed accessions. At each position of a given gene, accessions with homozygous and heterozygous non‐synonymous SNPs resulting in an amino acid change were given a score of 2 and 1, respectively. Lack of SNPs resulted in a score of 0. A ‘total non‐synonymous value’ (TNSV) was defined either as the sum of non‐synonymous values (T) at a given position of a single *BnSFAR* gene or as the sum of all non‐synonymous values of all *BnSFAR* genes. *n*: the number of accessions used for the calculation of mean SOC. The *P* value indicates the significance of pairwise comparisons.

We screened the genomes of 870 rapeseed accessions for SNPs within the twelve paralogs of five *BnSFAR* gene families using our re‐sequencing data (Wu *et al.*, 2019) and the ‘Darmor*‐bzh*’ sequence as the reference genome. Moreover, the SOC and FA composition of all accessions were determined (Dataset S1). For statistical analysis, homozygous SNP (+/+), heterozygous SNP (+/−) and lack of SNPs (−/−) at a certain position within a *BnSFAR* gene were given a 2, 1 and 0 score value, respectively. Only non‐synonymous SNPs were considered for the analysis. A ‘total non‐synonymous value’ (TNSV) was defined either as the sum of non‐synonymous values at a given position of a single *BnSFAR* gene or as the sum of all non‐synonymous values of all *BnSFAR1‐BnSFAR5* genes. As a result, accessions with TNSV larger than 20 had significantly higher average SOC than accessions with TNSVs < 20 (Figure 2, Dataset S1). However, there was no significant difference between accessions if TNSVs were between 10 and 20 or lower than 10 (Figure 2). We also examined the effect of SNPs on SOC for each *BnSFAR* gene subfamily separately (Dataset S1). We found significant effects of SNPs within *BnSFAR1* and *BnSFAR4* families, respectively (Figure 2). To investigate the role of SNPs in varying FA composition (*SFAR footprint*), we analysed the seed oleic acid (C18:1) content (OAC) (Dataset S1). Accessions with a TNSV higher than 20 had significantly lower OAC as compared to accessions with a TNSV < 20, indicating a SFAR loss‐of‐function effect (Figure S2). However, no significant difference was obvious if TNSVs were between 10 and 20 or if TNSVs < 10 (Figure S2). Studying SNP effects on the individual gene families resulted in significantly different OAC for *BnSFAR1* (*P* = 4.9e‐17), *BnSFAR4* (*P* = 2.3e‐5) and *BnSFAR5* (*P* = 0.0024) paralogs (Figure S2).

### Selecting *BnGDSL* genes expressed in developing seeds

**Figure 3:**
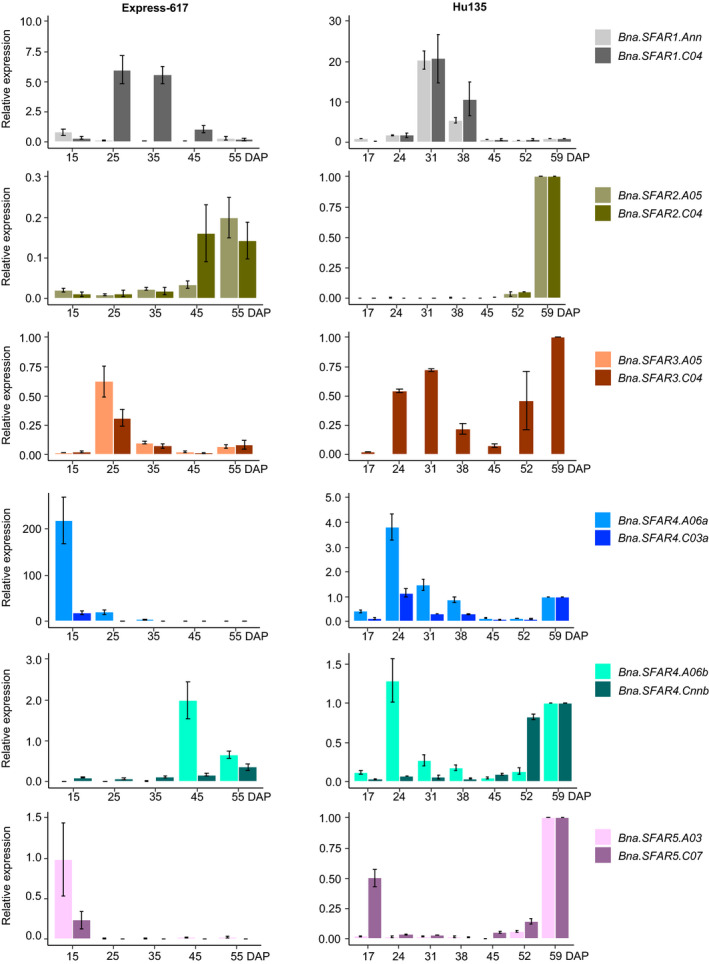
Relative expression of *BnSFAR1‐BnSFAR5* genes. Seeds from winter rapeseed variety Express‐617 were harvested 15, 25, 35, 45 and 55 days after pollination (DAP). Seeds from the Chinese semi‐winter‐type variety ‘Hu135’ were harvested 17, 24, 31, 38, 45, 52 and 59 DAP. Five biological samples for Express‐617 and three biological samples for Hu135 together with three technical replicates were used for analysis. Gene expression was quantified relative to *BnActin*. Error bars were defined by the SEM of five or three biological samples for Express‐617 and Hu135, respectively.

We sequenced the transcriptomes of developing seeds 16 and 40 days after pollination (DAP) (Zhou *et al.*, 2017). In total, 105 putative *BnGDSL* genes were found to be expressed in developing seeds: 75 of them were *AtGDSL* orthologs, while the rest did not display sufficient homology (FPKM, fragments per kilobase per million >1) (Figure S1, Dataset S2). Among the 75 *AtGDSL* orthologs, 14 genes were equally expressed at both developmental stages, while 22 and 39 genes were up‐regulated and down‐regulated, respectively, at 40 DAP (relative to 16 DAP) (Dataset S2). To verify the RNA‐seq data, we measured the relative expression of *BnSFAR1‐BnSFAR5* by RT‐qPCR at five developmental stages in the German winter‐type cultivar ‘Express‐617’ (15, 25, 35, 45 and 55 DAP) and seven developmental stages in the Chinese semi‐winter‐type cultivar ‘Hu135’ (17, 24, 31, 38, 45, 52 and 59 DAP) (Figure 3, Table S2). The results can be summarized as follows: (i) the RT‐qPCR results were generally in line with the transcriptome data. (iii) Genes from *BnSFAR4* subfamily‐a were highly expressed during early stages of seed development (15‐35 DAP), while *BnSFAR4* subfamily‐b genes were highly expressed at seed maturation and desiccation stages (45‐55 DAP). (iii) Also, *BnSFAR1* was highly expressed at early stages (25 and 35 DAP), but then their transcriptional activities dropped towards seed ripening (Figure 3). Only minor differences were obvious between winter and semi‐winter‐type cultivars.

### *SFAR* knockout mutants by CRISPR‐Cas and induced mutagenesis

We selected two *BnSFAR1* and four *BnSFAR4* genes because (i) non‐synonymous SNPs within these genes had a significant effect on SOC; (ii) they were differentially expressed between early and late stages of seed development; and (iii) their highest expression was between 35 and 55 DAP.

**Figure 4:**
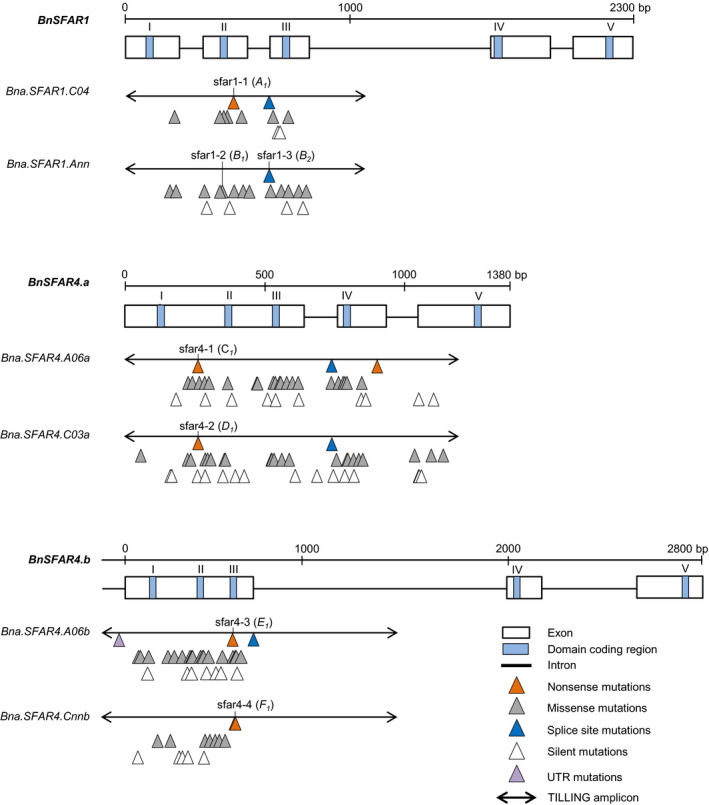
Location of EMS‐induced mutations in six *BnSFAR* genes belonging to three subfamilies, *BnSFAR1*, *BnSFAR4* subfamily‐a and *BnSFAR4* subfamily‐b. Mutations are displayed only if they were confirmed, after Sanger sequencing of M_3_ plants. The five GDSL consensus domain blocks are indicated by roman letters (I–V). The mutants are named ‘sfar’, and the mutant alleles are written in brackets.

Screening of 3,488 M_2_ families from our EMS TILLING resource revealed 163 mutations in *BnSFAR1* and *BnSFAR4* genes, corresponding to an average mutation frequency of 1/24.5 kb (Table S3). We identified non‐sense mutations for five paralogs, except for *Bna.SFAR1.Ann* where we found a mis‐sense mutation (sfar1‐2) in a strictly conserved SGNH amino acid motif and one splice site mutation (sfar1‐3) (Figure 4, Table S3, Table S4). We chose seven EMS single mutants for phenotyping. Of these, six mutants were used as parents to produce double mutants (Table S5). Because EMS causes a high number of genome‐wide random mutations, reduction of the background mutation load is vital. Therefore, we backcrossed mutants with the EMS donor plant Express‐617 (Figure S3a) and used allele‐specific markers (Table S2) to isolate homozygous mutant and wild‐type plants among segregating F_2_ and BC_1_F_2_ populations.

In polyploid species with high gene redundancy, single‐gene mutations usually do not have a phenotypic effect. Therefore, we crossed single *BnSFAR1* and *BnSFAR4* mutants to produce double mutants to measure SOC. We crossed either M_3_ single mutants directly (M_3_ × M_3_) or single mutants backcrossed once with the EMS donor Express‐617 (M_3_‐Express‐617 × M_3_‐Express‐617) (Figure S3b, Table S5).

**Figure 5:**
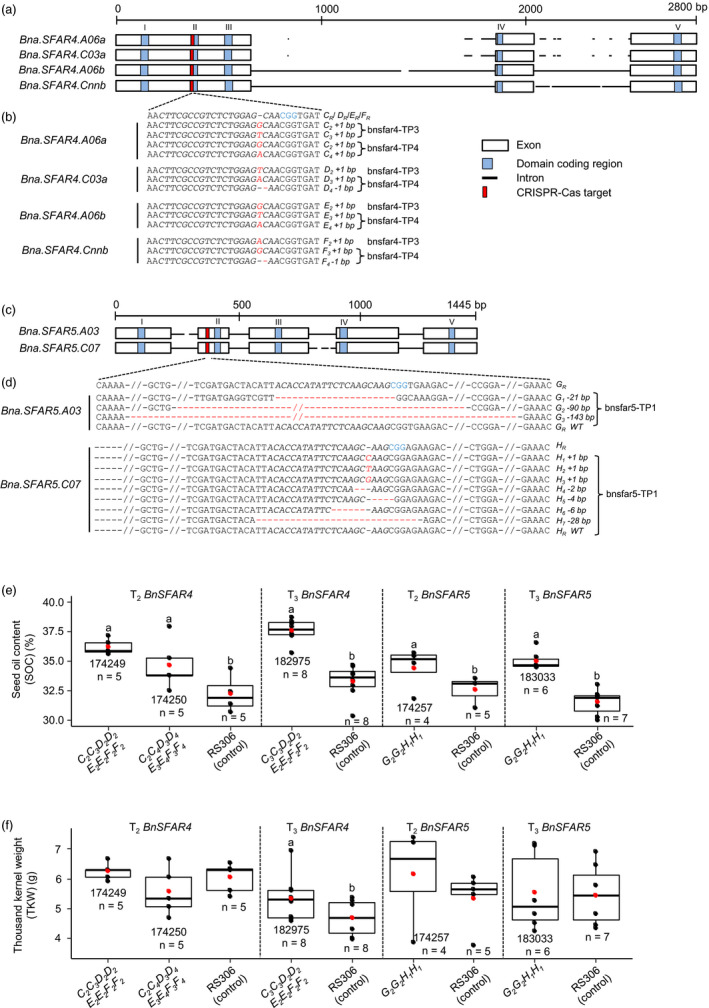
CRISPR‐Cas induced mutations in six *BnSFAR* genes and phenotypic analysis of *BnSFAR4* and *BnSFAR5* CRISPR‐Cas mutants. (a) A conserved 20 bp target was selected residing within domain coding block II of *BnSFAR4*. (b) Target sites were sequenced from regenerated plants after *Agrobacterium* transformation (T_1_ generation). Thirteen mutant alleles were identified in T_1_ plants named bnsfar4‐TP3 and bnsfar4‐TP4, and two of them are identical (*C_2_*) in both plants. (c) Gene structure of *BnSFAR5* genes. A conserved 20 bp region was targeted on exon 2 upstream of domain coding block II of *BnSFAR5*. (d) CRISPR‐Cas induced mutations in the T_1_ plants named bnsfar5‐TP1. The protospacer‐adjacent motif (PAM) is written in blue letters, while red colour indicates insertions and red dashes (‘‐’) indicate deletions. Mutant alleles are written left of the sequences. Wild‐type alleles are carrying a suffix ‘R’. (e) Seed oil content (SOC) and (f) thousand kernel weight (TKW) in T_3_ and T_4_ seeds of *BnSFAR4* and *BnSFAR5* mutants. Of each genotype, a minimum of four plants was studied. Seed codes and the number of plants used for phenotyping are written below the boxes. Oil extraction was done using n‐hexane micro‐extraction. One‐way ANOVA test was performed at *P < *0.05, and grouping was done using a Tukey test at *P < *0.05 for *BnSFAR4* T_3_ mutants, and an unpaired *t*‐test at *P* < 0.05 was performed for *BnSFAR4* T_4_ and *BnSFAR5* mutants. Within each phenotyping experiment (separated by dotted lines), different letters (a, b) indicate significant differences. The red dot in the box plot shows the mean SOC or mean TKW.

Next, we produced mutants by CRISPR‐Cas9‐induced mutagenesis to get multiple gene knockouts simultaneously. We searched for conserved sequences as target sites between four *BnSFAR4* and two *BnSFAR5* genes, separately. Only target sequences with no expected off‐target effects were considered (Figure S4). Finally, we chose target sites from exon 1 and exon 2 located within (*BnSFAR4*) or 19 bp upstream (*BnSFAR5*) of domain block II (Figure 5a,c). To knockout the *BnSFAR1* paralogs, we chose a target site from exon 1 next to the domain block I. The pCas9‐TPC construct was introduced into the winter rapeseed line RS306 by *Agrobacterium*‐mediated hypocotyl transformation (Braatz *et al.*, 2017). Transformation of 857 (*BnSFAR1*), 442 (*BnSFAR4*) and 754 excised hypocotyls (*BnSFAR5*) yielded two, five and two transgenic plants (Table S6), respectively, equivalent to transformation efficiencies between 0.2% and 1.1%.

We selected two primary T_1_ *SFAR4* transformants (bnsfar4‐TP3 and bnsfar4‐TP4) and the *SFAR5* (bnsfar5‐TP1) transformant for further studies. As an initial mutation screening, PCR products enclosing the target region were sequenced from T_1_ plants, and we found complete gene editing in all *BnSFAR4* paralogs (Figure 5b). We identified 13 different mutant alleles with single nucleotide insertions and deletions. Two were identical and were assigned the same allele identities (*C_2_*) (Figure 5b and Table S4). No wild‐type sequence could be detected. Each mutation resulted in a premature stop codon leading to a truncated protein. Hence, we can assume that all are loss‐of‐function mutations. In contrast, none of the T_1_ plants transformed with the *BnSFAR1* construct displayed any mutation within the target sequence.

Sequencing three *in vitro* clones of the *SFAR5* transformant bnsfar5‐TP1 (bnsfar5‐CP1, bnsfar5‐CP2 and bnsfar5‐CP3) revealed mutations in both *BnSFAR5* paralogs (Figure 5d and Table S4). Three mutations were 1 bp insertions three base pairs upstream of the PAM site, and the other ones were deletions (2–143 bp) between 283 and 423 bp from the start codon of the *BnSFAR5* genes. Compared to the *BnSFAR4* plants, wild‐type alleles were found in all clones, indicating their chimerism (Figure 5d and Table S4).

We analysed the inheritance of CRISPR mutations by genotyping T_2_ plants (Table S7). All *SFAR4* mutations found in the T_1_ plants were confirmed in the T_2_ generation, demonstrating that the parents were non‐chimeric, and mutations were stably inherited because each locus under study segregated for both mutant alleles detected in the T_1_ plants. Taking all four *BnSFAR4* loci together, 81 different T_2_ genotypes were expected. For better understanding, we examined the segregation pattern of alleles separately for each gene. In the case of bnsfar4‐TP4 (seed code 174250), segregation patterns of all four paralogs were consistent with Mendelian segregation (Table S7). Interestingly, the homozygous mutations in bnsfar4‐TP3 were 100% transmitted to the next generation giving rise to a stable mutant line (Table S7).

Then, we genotyped *BnSFAR5* T_2_ offspring of T_1_ clones. As expected, the T_2_ generation was segregating for multiple alleles at both loci. Therefore, we analysed the T_3_ generation to select homozygous double mutants. We did not observe Mendelian segregation in transgene of bnsfar5‐CP2 (seed code 174257) and bnsfar5‐CP3 (seed code 174258). However, mutant genes segregated accordingly (Table S7). Moreover, the absence of transgene‐free offspring in the investigated plants suggests the integration of more than one transgene during transformation.

### Mutants with multiple gene knockouts display increased seed oil content

We performed growth experiments with homozygous *SFAR* mutants to measure yield parameters. We selected two T_2_ families (174249 and 174250) and one T_3_ line (182975), which were fixed for the mutant alleles. Plants were grown in the greenhouse together with RS306 controls. The plants showed normal growth, flowering and seed set when compared to wild‐type RS306. In *BnSFAR4* mutants, SOC in T_3_ and T_4_ seeds was significantly increased by 9.7–14.5% and 12.9%, respectively, when compared to the donor line RS306 (Figure 5e and Table S8), whereas the TKW was not altered in T_3_ seeds, but significantly increased in T_4_ seeds (182975) (Figure 5f). Likewise, the *BnSFAR5* double mutants displayed a significant seed oil increase by 10.4% and 11.2% in T_3_ (174257) and T_4_ (183033) seeds, respectively (Figure 5e and Table S8). Their TKW and overall plant performance were not significantly different compared to RS306 (Figure 5f and Table S8). The erucic acid content was slightly reduced in two *BnSFFAR4* lines (174249 and 174250) when compared to RS306, which is a non‐quality synthetic rapeseed line. However, the reduction was only significant in 174249 in combination with a non‐significant increase in C18:1 and C18:3 contents, while in 174250 only C18:1 content was increased (Figure S5).

**Figure 6:**
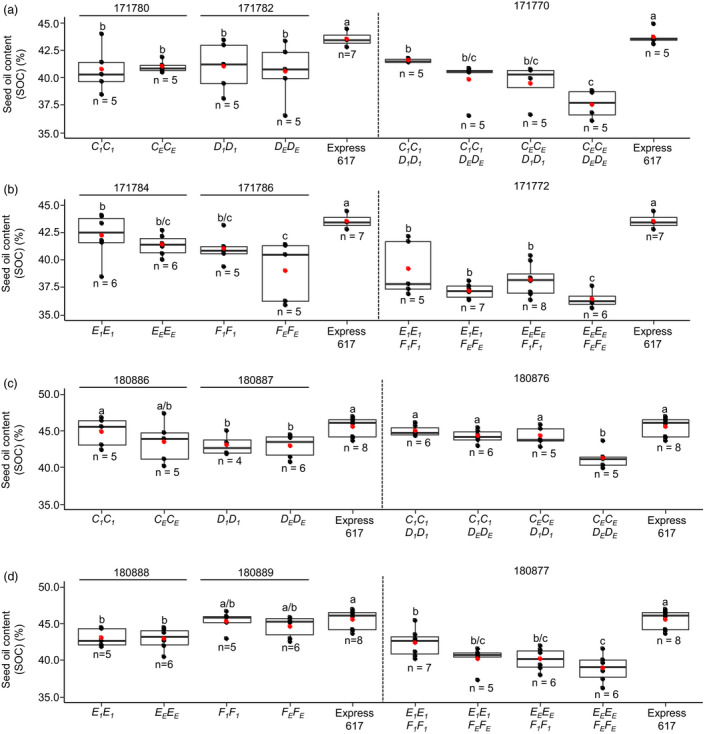
Seed oil content of segregating *BnSFAR4* EMS mutant progenies. (a) and (b) Seed oil content of F_2_ populations segregating for single mutations (171780, 171782, 171784 and 171786) and mutant progenies derived from M_3_ × M_3_ crosses (171770 and 171772) (see Table S5). (c) and (d) Seed oil content of BC_1_F_2_ (180886–180889) plants and mutant progenies from (M_3_ × Express‐617) × (M_3_ × Express‐617) crossing generations (180876 and 180877). One‐way ANOVA test was performed at *P* < 0.05, and grouping was done using the Tukey test at *P* < 0.05. The red dot in the box plot shows the mean SOC. Within each phenotyping experiment (experiments separated by dotted lines), different letters (a, b, c) indicate significant differences.

Then, we studied the effect of *SFAR* mutations in EMS mutant lines. The plant material included six segregating F_2_ populations from crosses between homozygous M_3_ plants (M_3_xM_3_) and Express‐617 backcross generations ((M_3_ × Express‐617) x (M_3_ × Express‐617)), and backcross populations (F_3_ and BC_1_F_3_) with reduced mutation load (Table S5). All plants were genotyped with allele‐specific primer combinations (Table S2) to distinguish between heterozygous and homozygous genotypes. Therefore, single mutants, double mutants and EMS‐treated plants carrying the non‐mutated alleles with roughly equal background mutation load could be employed in this experiment (Table S5). We did not observe significant differences in SOC between homozygous single mutants and plants with the wild‐type allele (171780, 171782, 171784, 171786, 180886‐180889) (Figure 6), while the SOC was significantly higher in the non‐mutagenized Express‐617 (Figure 6a,b). Noteworthy, in the backcrossed plants, the SOC of homozygous single mutants (*C_1_C_1_* (180886) and *F_1_F_1_* (180889)) was significantly higher (44– 45 %) demonstrating the bias caused by the mutation load in early mutant generations (Figure 6c,d).

Because single mutants did not have elevated SOC, we analysed *BnSFAR1* double mutants homozygous for the mutant alleles (*A_1_A_1_B_1_B_1_*). In two independent populations, no significant differences were found compared to mutant offspring with the Express‐617 allele (*A_E_A_E_B_E_B_E_*) (Table S8). Conversely, SOC was increased by 12.1% (171770), 10.3% (171772), 8.9% (180876) and 8.7% (180877) in *BnSFAR4.a* and *BnSFAR4.b* double mutants as compared to the mutants with the Express‐617 alleles (Figure 6). One mutant line (180876) already reached the SOC of the non‐mutated Express‐617 line despite its high mutation load (Figure 6c).

### Loss of *SFAR* function impacts oil body size but not seed vigour

**Figure 7:**
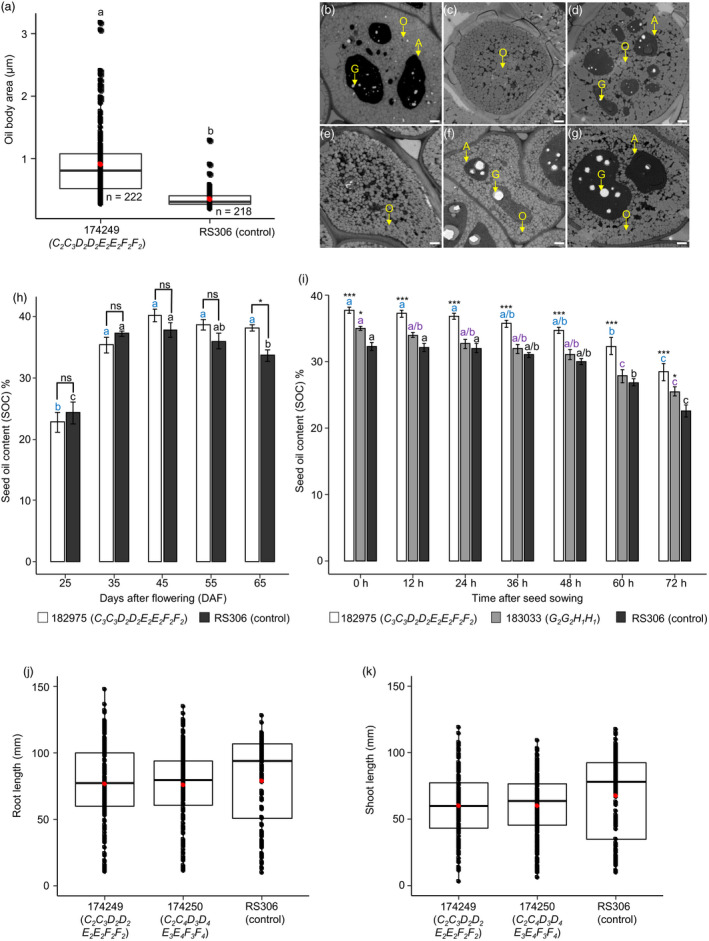
Analysis of seed oil bodies in T_3_ seeds, dynamics of seed oil accumulation and mobilization, and assessment of seed vigour in *BnSFAR* CRISPR‐Cas mutants. (a) Seed oil body area in seeds of the *BnSFAR4* mutant (174249) and seeds of RS306. Cross sections of cotyledons were analysed by transmission electron microscopy. An unpaired *t‐*test was performed at *P* < 0.05*.* Significance levels are indicated by letters. (b)‐(d) Oil bodies of *BnSFAR4* mutants and (e–g) RS306. Arrows indicate oil bodies (O), aleurone grains (A) and globoids (G). Bar = 1 µm. (h) The CRISPR‐Cas line 182975 (T_3_, *C_3_C_3_D_2_D_2_E_2_E_2_F_2_F_2_*) and RS306 were grown in the greenhouse, and seeds were harvested at different time points to measure SOC (*n* = 5). (i) SOC from germinating seeds at different time points after sowing (*n* = 6). (h) and (i) The n‐hexane micro‐extraction method was used for oil isolation. Data are presented as means ± SEM. One‐way and two‐way ANOVA were performed at *P* < 0.05, and grouping was done using the Tukey test at *P* < 0.05*.* Significance levels are indicated by blue (182975), purple (183033) and black letters (control) between different time points. Significant differences between mutants and controls are shown at *P* < 0.05 (*) and *P* < 0.001 (***) (j) Root and (k) shoot growth of two *SFAR* CRISPR mutants five days after sowing (DAS) (*n* = 124‐174). One‐way ANOVA test was performed at *P* < 0.05, and grouping was done using the Tukey test at *P* < 0.05.

We wanted to know why *SFAR* double mutants have higher SOC. We reasoned that cotyledon cells from developing seeds could contain altered oil bodies (OB) due to *SFAR* mutations resulting in elevated SOC. Therefore, OBs in mature seeds were investigated by transmission electron microscopy (TEM). We found that OBs from the CRISPR‐Cas mutant 174249 (*C_2_C_3_D_2_D_2_E_2_E_2_F_2_F_2_*) were significantly larger than OBs from RS306, indicating less degradation of TAGs during seed maturation (Figure 7a,b‐g).

Next, we investigated the effects of knockout mutations on oil accumulation during seed maturation. Seeds from T_3_ plants (CRISPR‐Cas mutant line 182975, *C_3_C_3_D_2_D_2_E_2_E_2_F_2_F_2_*) were harvested from 25‐55 DAP. The highest SOC was measured at 45 DAP in both mutant and RS306. The SOC dropped sharply in RS306, whereas a much slower decline was observed in the mutant (Figure 7h). These data confirm the physiological effect of *SFAR* mutations resulting in reduced oil degradation during seed maturation.

However, if seed oil mobilization is also retarded during germination, this could result in reduced seedling vigour. Therefore, we measured SOC at different stages after germination. As expected, oil content decreased with time in all genotypes (182975, 183033 and RS306) and however with an increased rate in RS306 (24.5% and 27% vs. 29.9%) (Figure 7i). It is well known that lipases are important in osmotic stress response. Therefore, we tested seedling growth of two CRISPR‐Cas mutants under mild stress conditions of 50 mm NaCl for five days. We found that *SFAR* mutations had no adverse effect on seed germination, which was in the range of the RS306 donor (Table S9). Moreover, root and shoot growth were not significantly different between mutants and RS306 (Figure 7j,k and Table S9).

## Discussion

Increasing SOC is a major focus in oil crop breeding. In previous years, the TAG biosynthesis pathway has been investigated intensively (Bates, 2016, Li‐Beisson *et al.*, 2013), and numerous studies have shown an elevation of SOC in rapeseed through the manipulation of transcription factors, FA transporters and promoters or inhibitors of TAG biosynthesis (Peng *et al.*, 2010; Tan *et al.*, 2011). During seed maturation, the balanced activities of seed oil synthesis and degradation genes ensure seed dormancy to prevent early sprouting, a process known as vivipary, which consumes seed reserves including the storage lipids (Wan et al., 2017). Our study provides the first successful example where knockout of *BnSFAR4* and *BnSFAR5* lipase genes in an oil crop resulted in higher SOC while germination rates and seed vigour remained unaffected.

We provide the first whole‐genome survey of all *BnGDSL* genes in rapeseed. The *BnGSDLs* from the A subgenome showed more diversity, in accordance with an overall higher genetic diversity within this subgenome. This is explained by the fact that *B. napus* × *B. rapa* crosses were more frequent than crosses with the other founder species, *B. oleracea* (Wu *et al.*, 2019). A candidate gene association study revealed that SOC was positively correlated with the number of non‐synonymous SNPs within the coding sequences of *BnSFAR* genes. Therefore, we propose to use natural variation from seed banks to search for new allelic variants within *GDSL* genes in rapeseed and other oilseed crops.

Less than 50% of the *BnGDSL* genes were expressed in developing seeds. The expression of *BnGDSL* genes may be affected by various endogenous and/or exogenous environmental stimuli. The expression profiles did not vary much between the winter and semi‐winter cultivar apart from four genes (*Bna.SFAR1.Ann, Bna.SFAR4.A06a*, *Bna.SFAR4.A06b* and *Bna.SFAR5.A03*). This could be explained by different upstream factors that regulate the expression of *BnGDSLs* as was found in *Arabidopsis* where the expression of *AtSFARs* was up‐regulated by gibberellin signalling (Chen *et al.*, 2012). The low transcriptional activity at later stages of seed development is probably the reason why *BnSFAR1* mutations did not affect SOC, although a minor effect from the mis‐sense mutation cannot be ruled out. Alternatively, these genes may have undergone neofunctionalization. Nevertheless, the knowledge of transcriptional profiles of *GDSL* genes is key to successful knockout experiments. Lipases initiate lipid mobilization by hydrolysing storage TAGs into glycerol and FAs to supply precursors for the β‐oxidation pathway during seed germination (Graham, 2008). Therefore, it was reasonable to investigate the effect of *BnSFAR* mutations on seed germination and seed vigour. It is noteworthy that the mutants did not differ in both characters from their donor genotypes.

We demonstrate that only multiple knockouts are useful in studying functionally redundant genes typical for polyploids. Although random mutagenesis was a common method to introduce new allelic variants into plant breeding, gene redundancy in rapeseed makes it challenging to reshape a trait by random mutagenesis (Braatz et al., 2018, Shah *et al.*, 2018). Conversely, CRISPR‐Cas‐mediated genome editing enables multiple mutations simultaneously. We used both random mutagenesis and site‐specific nucleases to induce mutations in *BnSFAR*s. As expected, we did not observe significantly increased SOC in single mutants compared to wild‐type plants. While pyramiding single mutations in one genotype is cumbersome and laborious, we obtained CRISPR‐Cas quadruple *BnSFAR4* knocked out mutants. In less than two years, stably inheriting homozygous T_3_ winter‐type mutant lines could be selected. As shown in previous studies (Yang *et al.*, 2018; Zhang *et al.*, 2019), we also found T_1_ plants with more than two alleles (haplotypes) at one locus as clear evidence for chimerism. A possible explanation is the low activity of the Cas9 nuclease caused by partial transgene silencing in the *BnSFAR5* T_1_ plant, which carries multiple transgene insertions. Gene silencing in transgenic plants with more than one copy of the transgene is reported for different plant species (Sohn *et al.*, 2011; Tang *et al.*, 2007). However, homozygous mutants could be easily selected in the T_3_ generation. The CRISPR‐Cas approach was conclusively more efficient than the TILLING approach.

Another shortcoming is the high number of background mutations after EMS mutagenesis. Considering mutations in six genes, each plant from our EMS TILLING population was found to carry ~46 000 mutations given the rapeseed genome size of 1130 Mb (Chalhoub *et al.*, 2014). Therefore, primary mutants suffer from a high mutation load, resulting in morphological and physiological anomalies. Hence, we reduced the mutation load by backcrossing with the non‐mutated donor. Then, the phenotypic effect in double mutants was assessed within segregating populations comparing homozygous genotypes (mutant and wild‐type allele) with equal mutation load. In our experiment with an F_2_ population segregating for *BnSFAR4.a* and *BnSFAR4.b* mutants, we found that the double mutants had a significantly higher SOC than homozygous genotypes with the wild‐type allele which aligns with our CRISPR‐Cas results where the same genes were mutated. Moreover, our results suggest additive action of *BnSFAR4* genes in rapeseed because only multiple mutations affected on SOC.

We used the synthetic rapeseed line RS306 for transformation experiments because explants displayed high shoot regeneration capacity. Typical for synthetic rapeseed, RS306 has a comparatively low SOC compared to modern varieties whose SOC content is in a range between 40 and 50%. There are two lines of evidence why we expect that modern varieties will also display elevated SOC after knockout of *BnSFAR4* and *BnSFAR5* genes. First, it has been reported that SOC equally declined during seed maturation in high and low SOC inbred lines (Shahid et al., 2019). Therefore, we think that the knocking‐out of *BnSFAR* genes will increase SOC even in modern varieties. Second, the variety Express‐617, which we used to screen EMS‐induced mutations, has a comparatively high SOC around 44%. We observed that SOC was significantly higher in *BnSFAR4* double mutants in comparison with the wild‐type plants with the same mutation load. Even with a high mutation load, one double mutant (180876) reached the SOC of the non‐mutated Express‐617 line. These results provide evidence that knockout of *BnSFAR* genes in an elite line will evenly increase SOC.

What are the consequences of *SFAR* knockout mutations on the cellular and physiological level? In our study, *BnSFAR4* mutants had larger OBs in comparison with the donor RS306. In this respect, ambiguous data have been published in the past. Siloto *et al. *(2006) demonstrated that the knockout of oleosin genes caused unusually large OBs, which correlated with lower SOC. Contrastingly, in another study OB size in rapeseed was positively correlated with SOC (Tan *et al.*, 2019) where embryos were investigated from 21, 28 and 35 DAP. We reason that the bigger size of OBs in our *BnSFAR4* mutant was due to reduced TAGs decomposition, which has also been observed in previous research in *Arabidopsis* (Chen *et al.*, 2012).

Perspectives for practical breeding largely depend on the background mutation load and legal scenarios. The high number of background mutations in the EMS mutants is a shortcoming. Therefore, numerous backcrossings are needed to develop a mutant line with enhanced SOC and reduced background mutation load. While backcrossing with winter rapeseed is time‐consuming because of the vernalization requirement, the use of spring rapeseed as backcross parent has been proposed to accelerate generation cycles. We are currently producing *BnSFAR4* double mutants by backcrossing with an early flowering spring rapeseed in combination with genomic background selection using SNP arrays. This highlights the opportunity to reduce background mutations resulting from EMS mutagenesis with a speed breeding protocol (Watson *et al.*, 2018).

In this way, CRISPR‐Cas mutagenesis is clearly superior, because the desired mutation can be directly incorporated into an elite genome. Moreover, multiple mutations can be achieved in a single plant as demonstrated by this study. However, the application of CRISPR‐Cas‐induced mutations in plant breeding is largely hampered especially in the European Union, due to the current legislation where these plants are classified as genetically modified organisms (GMOs) (Kupferschmidt, 2018). In contrast, EMS mutants are not considered as GMO and can thus be used by breeders without legal constraints. Therefore, we expect that the *BnSFAR* EMS mutants will be effectively used in Europe, whereas the CRISPR‐Cas mutants will be preferred in states with less restrictive legislation like North America or Australia.

To conclude, our results not only shed light on the understanding of seed oil degradation in a polyploid oil crop but also open a new path for breeding for higher SOC. *BnSFAR* mutants will enable increased rapeseed oil yield per unit area, which is an important aim in many (developing) countries such as India, China or Bangladesh which largely depend on seed oil import. Moreover, we propose the application of *SFAR* knockout in other oil crops.

## Experimental procedures

### Identification of *GDSL* genes in the rapeseed genome

Sequences and chromosome positions of annotated *GDSL* (*AtGDSL*) genes in *Arabidopsis* were obtained from the TAIR database (https://www.arabidopsis.org/). *BnGDSLs* in the rapeseed genome were identified searching for genes with the IPR001087 domain (http://www.ebi.ac.uk/interpro/entry/IPR001087) in rapeseed (Genoscope‐INRA unmasked v4.1) with an e‐value below 1E‐30 using InterProScan 5.36‐75.0 (http://www.ebi.ac.uk/interpro/) (Jones *et al.*, 2014), and some of them were ruled out (Dong *et al.*, 2016). The sequences and locations of *BnGDSLs* were gained from the rapeseed Genome Browser in GENOSCOPE (http://www.genoscope.cns.fr/brassicanapus/). Visualization of collinear analyses among the *AtGDSLs* and *BnGDSLs* from the A and C subgenomes was performed using Circos (http://circos.ca/) (Krzywinski *et al.*, 2009). Multiple alignments were performed for nucleotide and amino acid sequences. The phylogenetic trees were constructed using the neighbour‐joining (NJ) method by ClustalW2 (http://www.clustal.org/clustal2/) and visualized in MEGA × 10.0.5 (http://www.megasoftware.net/) (parameters; 1000 bootstraps, Poisson model, Pairwise deletion) (Kumar *et al.*, 2018). The distribution of *BnGDSLs* across the rapeseed genome was visualized using Mapchart 2.32 (Voorrips, 2002) (https://www.wur.nl/en/show/Mapchart.htm).

### RNA‐seq and data analysis

Developing seeds from the rapeseed cultivar ‘ZY511’ at 16 and 40 DAP were harvested for RNA extraction. Total RNA was extracted using an RNA Extraction Kit (Omega Bio‐Tek, Norcross, GA, USA). RNA‐seq was performed by Biomarker Technology Co. (Beijing, China). Sequencing libraries were generated using the NEBNext Ultra RNA Library Prep Kit (NEB, Ipswich, MA) following the manufacturer’s manual. The libraries were sequenced on an Illumina HiSeq^TM^ 2500 platform, and paired‐end reads were generated. Clean reads were mapped to the rapeseed reference genome using Hisat2 software (Kim *et al.*, 2015). Quantification of transcription levels was estimated by fragments per kilobase per million (FPKM). Differential expression analysis of two samples was performed using the R package DESeq2 (Love *et al.*, 2014). The resulting *P* values were adjusted using Benjamini and Hochberg’s approach to controlling the false discovery rate (FDR). The parameters (FDR < 0.05 and |log_2_^(fold change)^| ≥ 1) were set as the thresholds for a significantly different expression.

### Expression analysis by RT‐qPCR

We used the winter rapeseed Express‐617 and the semi‐winter rapeseed Hu135. Express‐617 plants were grown under greenhouse conditions (16‐h light/8‐h dark, ~23‐24 °C), while Hu135 were grown in the field (Zhejiang University, Hangzhou) and marked for the pollination date. Seeds were harvested at 15, 25, 35, 45 and 55 DAP from Express‐617 and 17, 24, 31, 38, 45, 52 and 59 DAP from Hu135, shock‐frozen in liquid nitrogen and stored at −70 °C. We used ~ 50 mg of seeds for RNA isolation with the peqGold Plant RNA Kit (PEQLAB Biotechnologie GmbH, Erlangen, Germany) following the manufacturer’s instructions. The quality of the RNA was checked by agarose gel electrophoresis and a NanoDrop2000 spectrophotometer (Thermo Fisher Scientific, Waltham, MA). For the expression analysis, we used 2 µL of cDNA (5 ng/µL concentration) synthesized with the First Strand cDNA Kit (Thermo Fisher Scientific). Relative expression was measured using *BnACTIN2* and *BnACTIN7* as internal controls for Express‐617 and Hu135, respectively (Table S2). The difference between the cycle threshold (*C*_t_) of target genes and the Ct of the control gene ( $\Delta C_{t}\;=\;C_{t_{\text{target}\;\text{gene}}}-C_{t_{\text{control}}}$) was used to calculate the normalized expression of target genes.

### Searching for SNPs within *BnSFAR* genes

A total of 870 rapeseed genomes were sequenced in our previous project (Wu *et al.*, 2019). All clean reads for each accession were mapped to the ‘Darmor*‐bzh*’ genome (v4.1 genome, http://www.genoscope.cns.fr/ brassicanapus/data/) using the MEM algorithm of Burrows‐Wheeler Aligner (Li and Durbin, 2009) (BWA v0.7.5a‐r405). The mapping results were processed by sorting and duplicate reads marking SAMTOOLS (Li *et al.*, 2009) (v1.1) and PICARD (http://broadinstitute.github.io/picard/;v1.94). SNPs were called by the HaplotypeCaller module in GATK and were filtered with the parameters (QD < 2.0 || MQ < 40.0 || FS> 60.0 || QUAL < 30.0 || MQrankSum < −12.5 || ReadPosRankSum < −8.0 –clusterSize 2 –clusterWindowSize 5). The SNPs identified by GATK were further filtered; only the SNPs with a minor allele frequency >5% and <50% were considered as high‐quality SNPs. The SNP annotation was performed based on the rapeseed v4.1 genome using the snpEff software (Cingolani *et al.*, 2012). SNPs within exons of annotated *BnSFAR* genes were classified as synonymous SNPs and non‐synonymous SNPs, and the cumulative effects of non‐synonymous SNPs were calculated as TNSV.

### CRISPR‐Cas mutagenesis and mutant detection

We selected conserved 20 bp targets within exons adjacent to the NGG PAM site separately for *BnSFAR4* and *BnSFAR5*. A BLAST search against the rapeseed reference genome (Darmor*‐bzh* version 4.1) was performed to identify putative off‐targets. We used the binary vector system, pChimera and pCas9‐TPC for *Agrobacterium*‐mediated transformation following the protocol previously published (Fauser *et al.*, 2014). The final pCas9‐TPC was transformed into the *A. tumefaciens* strain GV3101 pMP90RK for plant transformation.

Rapeseed hypocotyls of the winter‐type RS306 were transformed following the protocol described previously (Zarhloul *et al.*, 2006) with minor modifications. Here, we applied 400 mg/L ticarcillin and clavulanate for the elimination of *Agrobacterium* and 5 mg/L of phosphinotricin for transgenic plant selection. Leaf genomic DNA was isolated from transgenic plants using the standard CTAB method. The presence of the transgene was confirmed after performing PCR using Cas1‐F and Cas1‐R primers (Table S2). We also cloned PCR products amplified with paralog‐specific primers from T_1_ plants into the pGEM‐T vector and transformed them into *Escherichia coli* (DH5α cells, DNA Cloning Service, Hamburg). Single colonies were picked for PCR, and mutations were identified by Sanger sequencing using CLC Main Workbench version 7.6.4 (CLC bio, Aarhus, Denmark).

### EMS mutant screening by TILLING

We screened 3,840 M_2_ plants of our EMS‐mutated winter rapeseed Express‐617 TILLING population for mutations within *BnSFAR* genes. Paralog‐specific primers were developed based on the reference genome Darmor‐*bzh* (version 4.1), and specificity was confirmed after Sanger sequencing (Table S2). We amplified pooled M_2_ plant DNA using the primers labelled with infrared fluorescent dyes (IRD) for mutation screening following the protocol previously described (Till *et al.*, 2006). Subsequently, we checked samples on agarose gels (1%, 100 V, 10 min) for quality. Pools with expected amplicon size were allowed for heteroduplex formation, followed by *Cel*I nuclease digestion. Samples were purified using Sephadex dry G‐50 powder (GE Healthcare, Chicago, IL) and separated by polyacrylamide gel electrophoresis on a LICOR 4300 DNA analyser (https://www.licor.com). GelBuddy software was used to identify mutations (Zerr and Henikoff, 2005).

### Plant materials and growth conditions

The re‐synthetic winter rapeseed RS306 was used for *Agrobacterium*‐mediated plant transformation. Surface sterilized seeds were germinated under low‐light conditions for 5–7 days at 24 °C, and hypocotyl explants of 0.5–1.0 cm were used for plant transformation. Rooted T_1_ plants were transferred into the greenhouse after acclimation. T_1_, T_2_ and T_3_ plants were grown in 11 × 11 cm pots in the greenhouse (16‐h light/8‐h dark at ~23–24 °C) together with RS306 control. All plants were vernalized for ten weeks (16‐h light/8‐h dark at 4 °C).

Selected M_3_ EMS mutants were crossed and backcrossed with Express‐617 to produce segregating F_2_ and BC_1_F_2_ progenies. We also crossed M_3_ single mutants to produce an F_2_ population to select homozygous double mutants and wild types (Table S5). All plants were grown in 11 × 11 cm pots under greenhouse conditions along with non‐mutated Express‐617 controls. Plants were vernalized for eight weeks. Plants selected for selfing were bagged before flowering to prevent cross‐pollination and fertilized with 0.5 g of Compo Blaukorn Classic universal fertilizer (Compo, Münster, Germany) before flowering.

### Genotyping and phenotyping of mutant plants

Allele‐specific markers were developed to genotype segregating EMS mutant progenies. For each paralog, we used a paralog‐specific primer pair along with either a mutant‐ or wild type‐specific primer pair (Table S2). After genotyping with allele‐specific primers, only the plants homozygous either for mutant or for wild‐type alleles were Sanger sequenced to verify the mutations. Plants grown under greenhouse condition were used for phenotyping. Important agronomic traits like seed number, seed weight and plant height were recorded on a single plant basis. Seeds were aliquoted (50‐100 seeds/plant) for seed oil extraction, and these samples were cooled in liquid nitrogen immediately and stored at −70 °C until use.

### Oil and fatty acid measurements

According to standard oil extraction protocols (Manirakiza *et al.*, 2001), an *n*‐hexane micro‐extraction method was developed adjusting to the small sample sizes in our experiments. We used 50–70 mg of finely milled seed powder per sample and extracted the oil twice with 1 mL of n‐hexane. During the first round, seed powder was weighed into a 2‐ml glass vial and extracted for 16 h with 1 mL of *n*‐hexane at room temperature in an overhead shaker. The sample was centrifuged at 4000 g for 10 min, and the n‐hexane supernatant was transferred into a new vial. The pellet was extracted for a second time with 1 ml n‐hexane for one hour and centrifuged as written above, and the supernatants of both extracts were combined. The solvent was removed in a rotary evaporator at 40°C (Bachofer vacuum concentrator) for 2 h. Total SOC was measured by weighing and calculated in per cent on a dry weight basis. The extracted oil was stored at −20 °C and used for the FA profile measurements using gas chromatography (Chen *et al.*, 2012). SOC and the seed FA composition were determined in 870 rapeseed accessions using near‐infrared spectroscopy (ANTARIS II, Thermo Scientific™, WI, USA). Three biological replicates of each accession were measured.

### Germination and seedling vigour tests

For seed germination tests, we used T_3_ CRISPR‐Cas mutant seeds. Seeds were sown on ½ MS medium containing 50 mm NaCl and grown in the dark at room temperature. Plates were kept at a 15° vertical angle in the dark. Plates were opened daily for ca. one minute to take photographs, and ImageJ software (https://imagej.net/) was used to analyse root and shoot length five days after sowing.

### Oil accumulation and mobilization

For the oil accumulation test, we harvested seeds (100–150 mg) from *BnSFAR4* T_3_ mutants and RS306 controls at 25, 35, 45, 55 and 65 DAP. Plants were grown under greenhouse conditions. For the seed oil mobilization test, T_4_ *BnSFAR4* mutant and wild‐type RS306 seeds were sown on wet Whatman filter paper, and samples were taken 0, 12, 24, 36, 48, 60 and 72 h after sowing. After sampling, seeds were shock‐frozen in liquid nitrogen and stored at −70 °C until oil extraction using n‐hexane micro‐extraction.

### Oil body analysis

OB size was measured in seeds from mutant and wild‐type plants. Fully mature seeds from three biological replicates of each genotype were selected for sectioning. Perpendicular transections were produced, and the sections with the largest oval‐shaped surface area were selected for quantification. Three cells located in the middle of a section were selected for the measurement of OB size. The cells were photographed using an OLYMPUS SZ 61 stereomicroscope (Tokyo, Japan). For the observation of oil bodies, electron micrographs were taken using a transmission electron microscope (TEM) (JEM‐1230, Tokyo Japan) (Chen *et al.*, 2012). The software ImageJ 1.52p (https://imagej.nih.gov/) was used to measure the surface of the oil bodies. The automatic measurements were improved and corrected by manual quantification.

### Statistical analysis

Statistical analysis was performed by one‐way analysis of variance (ANOVA), two‐way ANOVA, Tukey’s test or two‐tailed unpaired *t*‐test with GraphPad Prism (version 5.00 for windows): GraphPad Software, San Diego, CA, USA.

## Conflict of interest

The authors declare no conflict of interests.

## Author contributions

N.L.K and H.W planned the experiments, produced and analysed the data and involved in manuscript writing. H.H, L.J and C.J examined the data and were involved in manuscript writing.

## Supporting information


**Figure S1** Distribution of GDSL genes across the rapeseed subgenomes and their expression in developing seeds
**Figure S2 **The effect of non‐synonymous single nucleotide polymorphisms (SNP) within *BnSFAR* genes on oleic acid content in 870 non‐related rapeseed accessions.
**Figure S3 **Crossing schemes and pedigrees of plant materials used in this study.
**Figure S4 **Screening the rapeseed reference genome for putative *BnSFAR4* and *BnSFAR5* off‐target sequences.
**Figure S5 **Fatty acid profiles in T_3_ seeds of two *BnSFAR4* mutants and the RS306 control.
**Table S1 **Features of the *BnSFAR* genes used in this study.
**Table S2 **Primers used in this study.
**Table S3 **EMS‐induced mutations in *BnSFAR1* and *BnSFAR4* genes.
**Table S4 **EMS and CRISPR‐Cas mutations used for further studies.
**Table S5 **Production of EMS mutants by crossing M_3_ plants homozygous for the mutant allele.
**Table S6 **Results of the *Agrobacterium*‐mediated rapeseed hypocotyl transformation.
**Table S7 **Inheritance of CRISPR‐Cas mutations in *BnSFAR4* and *BnSFAR5*.
**Table S8 **Phenotyping data of EMS and CRISPR‐Cas *BnSFAR1*, *BnSFAR4* and *BnSFAR5* mutants.
**Table S9 **Seed germination, root, and shoot growth 5 DAS in T_3_ lines with *BnSFAR* knock‐out mutations and in RS306.

**Dataset S1** Seed oil content and oleic acid composition of all accessions

**Dataset S2** BnGDSL agene expression in developing seeds
