# Supplementary material for: Elevating seed oil content in a polyploid crop by induced mutations in SEED FATTY ACID REDUCER genes
Source: Plant Biotechnol J. 2020 Apr 13;18(11):2251–66. doi: 10.1111/pbi.13381 (PMC7589255; doi:10.1111/pbi.13381)
Supplement: Supplementary file 1 — Figure S1 Distribution of GDSL genes across the rapeseed subgenomes and their expression in developing seeds Figure S2 The effect of non‐synonymous single nucleotide polymorphisms (SNP) within BnSFAR genes on oleic acid content in 870 non‐related rapeseed accessions. Figure S3 Crossing schemes and pedigrees of plant materials used in this study. Figure S4 Screening the rapeseed reference genome for putative BnSFAR4 and BnSFAR5 off‐target sequences. Figure S5 Fatty acid profiles in T3 seeds of two BnSFAR4 mutants and the RS306 control. Table S1 Features of the BnSFAR genes used in this study. Table S2 Primers used in this study. Table S3 EMS‐induced mutations in BnSFAR1 and BnSFAR4 genes. Table S4 EMS and CRISPR‐Cas mutations used for further studies. Table S5 Production of EMS mutants by crossing M3 plants homozygous for the mutant allele. Table S6 Results of the Agrobacterium‐mediated rapeseed hypocotyl transformation. Table S7 Inheritance of CRISPR‐Cas mutations in BnSFAR4 and BnSFAR5. Table S8 Phenotyping data of EMS and CRISPR‐Cas BnSFAR1, BnSFAR4 and BnSFAR5 mutants. Table S9 Seed germination, root, and shoot growth 5 DAS in T3 lines with BnSFAR knock‐out mutations and in RS306. [file PBI-18-2251-s001.pdf]

Supplementary Information for

Elevating seed oil content in a polypoid crop by induced mutations in *SEED FATTY ACID REDUCER* genes

Nirosha L. Karunarathna[a], Haoyi Wang[b], Hans-Joachim Harloff[a], Lixi Jiang[b,1], Christian Jung[a,1]

[a]Plant Breeding Institute, Christian-Albrechts-University of Kiel, Olshausenstrasse 40, D-24098 Kiel, Germany

[b]Institute of Crop Science, Zhejiang University, Yu-Hang-Tang Road 866, Hangzhou, 310058, China

[1]Corresponding author name
Christian Jung.: c.jung@plantbreeding.uni-kiel.de
Lixi Jiang: jianglx@zju.edu.cn

Supplementary Figures

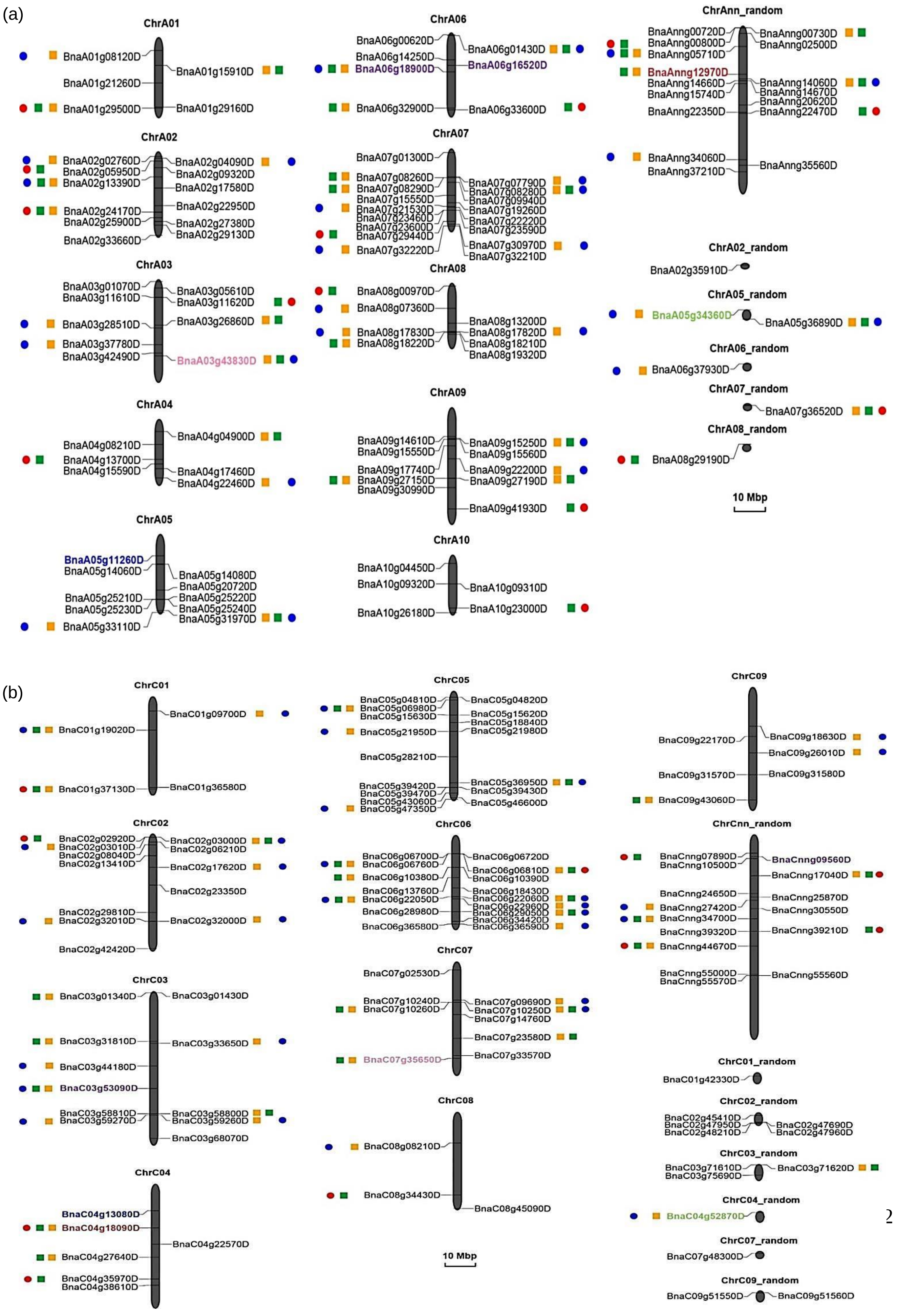

Figure S1: Distribution of *GDSL* genes across the rapeseed subgenomes and their expression in developing seeds. Altogether 111 *BnGDSL* genes were mapped to each of the two subgenomes A (a) and C (b). 'Chr.Ann' or 'Chr.Cnn' indicates unmapped sequences. Genes labeled with 'random' have unknown chromosome positions. The *BnSFAR1* to *BnSAFR5* genes are written in red, blue, green, purple and pink colors, respectively. The orange or green squares on the right and left sides of a gene indicate the gene expression as revealed by RNA-seq (FPKM>1) at 16 and 40 DAP, respectively. The red and blue dots indicate up- or down-regulation of the gene at 40 DAP relative to 16 DAP, as defined by the log2 expression folds >1 or <1.

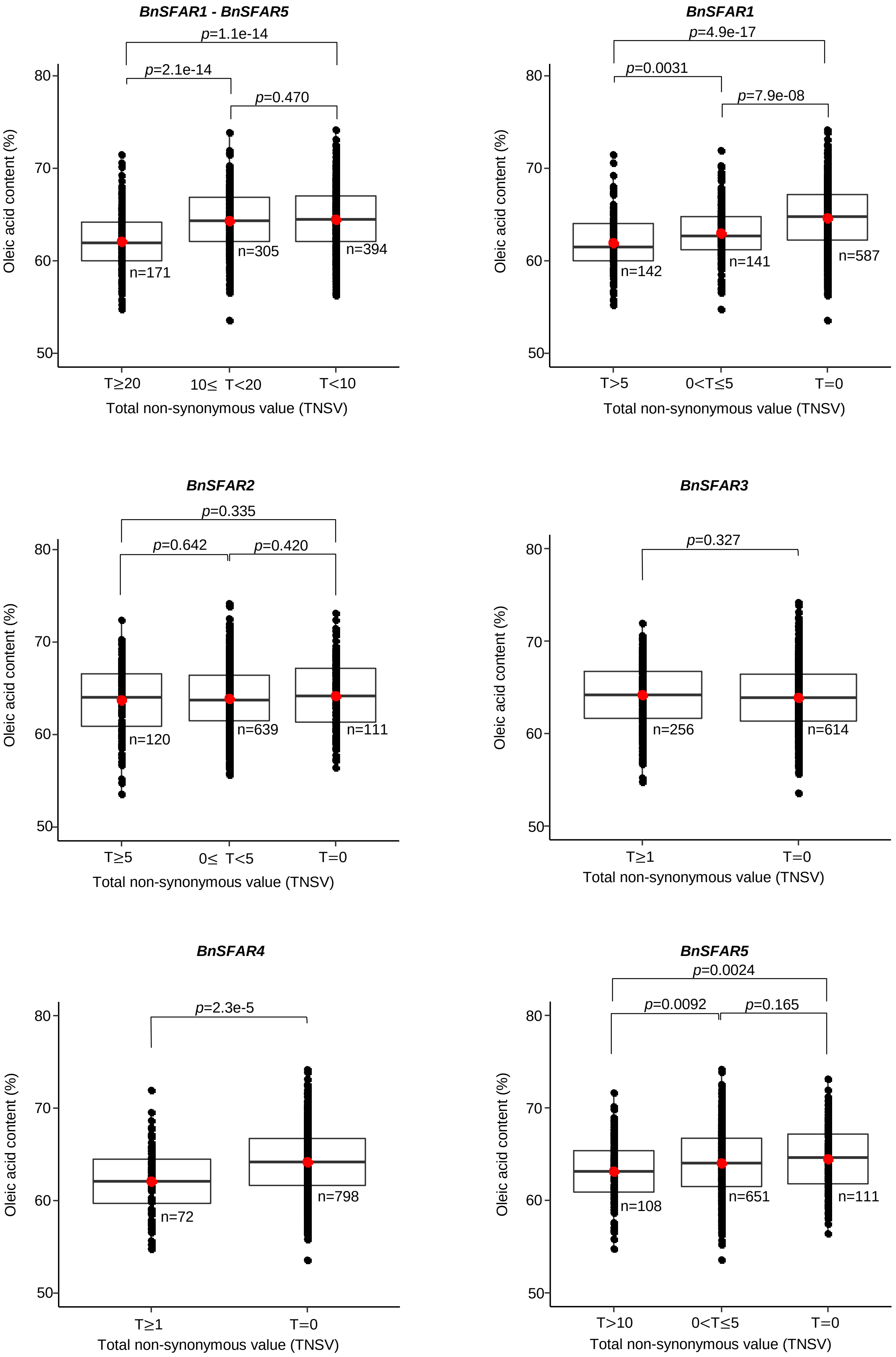
BnSFAR1 - BnSFAR5
p=1.1e-14
p=2.1e-14
p=0.470
n=171
n=305
n=394
T≥20
10≤ T<20
T<10
Oleic acid content (%)
Total non-synonymous value (TNSV)
BnSFAR1
p=4.9e-17
p=0.0031
p=7.9e-08
n=142
n=141
n=587
T>5
0<T≤5
T=0
Oleic acid content (%)
Total non-synonymous value (TNSV)
BnSFAR2
p=0.335
p=0.642
p=0.420
n=120
n=639
n=111
T≥5
0≤ T<5
T=0
Oleic acid content (%)
Total non-synonymous value (TNSV)
BnSFAR3
p=0.327
n=256
n=614
T≥1
T=0
Oleic acid content (%)
Total non-synonymous value (TNSV)
BnSFAR4
p=2.3e-5
n=72
n=798
T≥1
T=0
Oleic acid content (%)
Total non-synonymous value (TNSV)
BnSFAR5
p=0.0024
p=0.0092
p=0.165
n=108
n=651
n=111
T>10
0<T≤5
T=0
Oleic acid content (%)
Total non-synonymous value (TNSV)

Figure S2: The effect of non-synonymous single nucleotide polymorphisms (SNP) within *BnSFAR* genes on oleic acid content in 870 non-related rapeseed accessions. At each position of a given gene, accessions with homozygous and heterozygous (non-synonymous SNPs) resulting in an amino acid change were given a score of 2 and 1, respectively. Lack of SNPs resulted in a score of 0. A ‘Total Non-Synonymous Value’ (TNSV) was defined either as the sum of non-synonymous values at a given position of a single *BnSFAR* gene or as the sum of all non-synonymous values of all *BnSFAR1*- *BnSFAR5* genes. n: number of accessions used for the calculation of mean oleic acid content. The *p* value indicates the significance of pairwise comparisons.

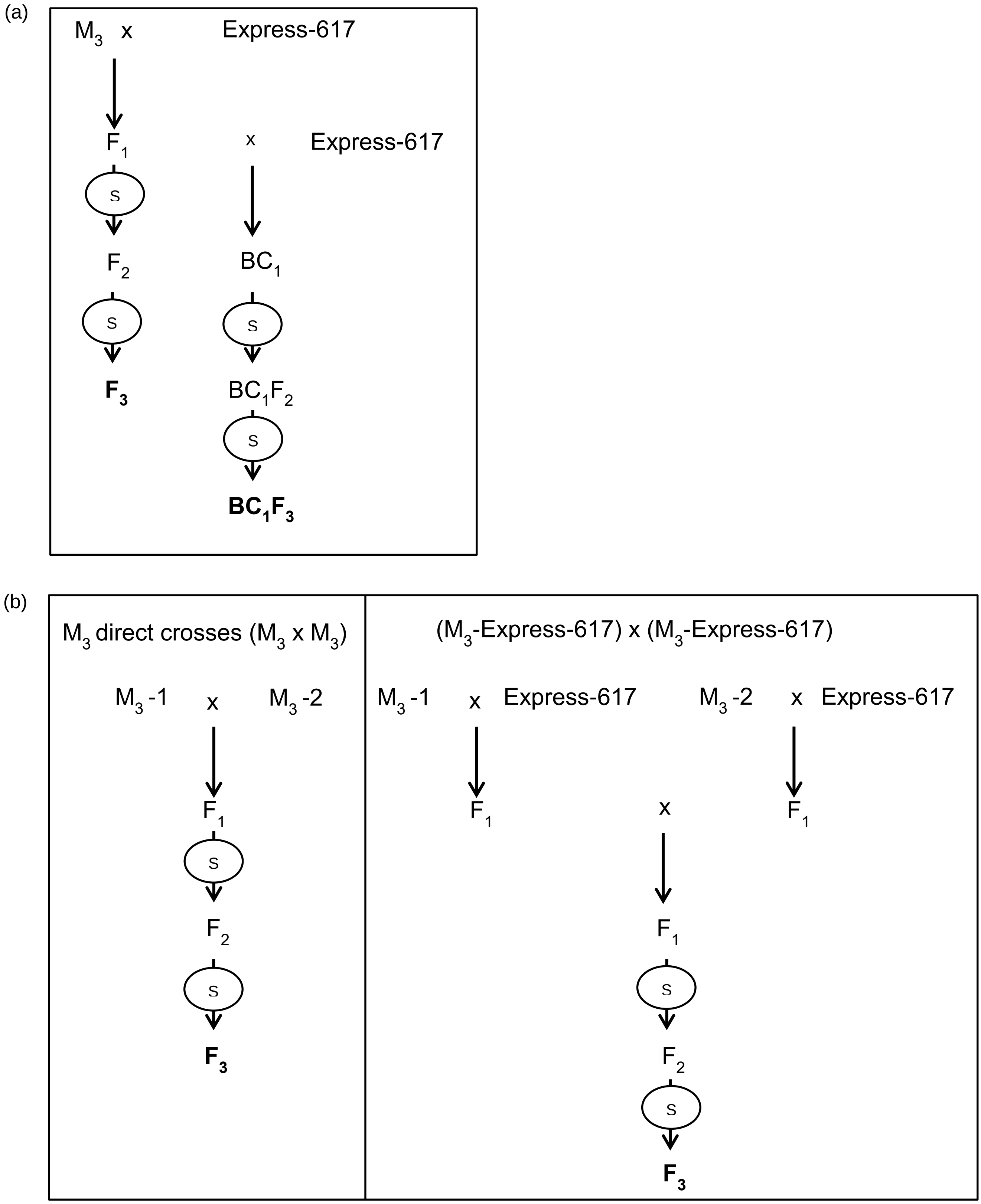

Figure S3: Crossing schemes and pedigrees of plant materials used in this study (a) M_3 single mutants were crossed and backcrossed with the EMS donor Express-617 to reduce mutation load. (b) M_3 single mutants were crossed to produce double mutants. Homozygous M_3 plants were either crossed directly with each other (M_3 x M_3) or they were first crossed with Express-617 (M_3-Express-617) x (M_3-Express-617).

```
Bna.SFAR4.CR2   cttcgccgtctctggagcaacgg

chrCnn_random   CTTCGCCGTCTCTGGAGCAACGG ←
       chrC03   CTTCGCCGTCTCTGGAGCAACGG ←
       chrC03   CTTCTCCGCCTCTGGAGCAAC
       chrC03     TCTCCATCTCTGGAGCAACG
       chrA06   CTTCGCCGTCTCTGGAGCAACGG ←
       chrA06   CTTCGCCGTCTCTGGAGCAACGG ←
       chrA10     TCGCCGTCTCTGGAGC
chrAnn_random     TCTCCATCTCTGGAGCAACG
       chrC09       GCCGTCTCTGGAGCA
       chrC09       GCCGTCTCTGGAGCA
       chrC08        CCGTCTCTGGAGCAA
       chrC06         CGTCTCTGGAGCAAC
       chrC01   CTTCGCCGTCTCTGG
       chrA09       GCCGTCTCTGGAGCA
       chrA09    TTCGCCGTCTCTGGA
       chrA07   CTTCGCCGTCTCTTTAGCAA
       chrA05   CTTCGTC-TCTCTGGAGCAACG
       chrA04     TCTCCATCTCTGGAGCAACG
       chrA04     TCTCCATCTCTGGAGCAACG
       chrA03     TCTCCATCTCTGGAGCAACG
       chrA03    TTCGCCGTCTCTGGA
```

```
Bna.SFAR5.CR5   acacc-atattctcaag-caagcgg

       chrC07   ACACC-ATATTCTCAAG-CAAGCGG ←
       chrA03   ACACC-ATATTCTCAAG-CAAGCGG ←
       chrC06      CC-ATATTCTCAAG-CAAG
       chrA02      CC-ATATTCTCAAG-CAAG
       chrC05      CC-ATATTCTCAAG-CAA
       chrC05     ACC-AAATACTCAAG-CAAGCG
       chrC05         ATATTCTCAAG-CAAG
       chrC05          TATTCTCAAG-CAAGC
chrCnn_random   ACACCTATATTCTCAAG-CAA
chrC03_random    CACC-ATATTCTCAAGACAAG
       chrC04      CC-ATATTCTCAAG-CA
       chrC04      CC-ATATTCTCAAG-CA
       chrC03       C-ATATTCTCAAG-CAA
       chrC02         ATATTCTCAAG-CAAG
       chrA07          TATTCTCAAG-CAAGC
       chrA05          TATTCTCAAG-CAAGC
```

Figure S4: Screening the rapeseed reference genome for putative *BnSFAR4* and *BnSFAR5* off-target sequences, which could be altered by CRISPR-Cas induced mutagenesis with the single guide RNAs used in this study. The 20 bp nucleotide sequences including the PAM (marked with blue line) sites were used to BLAST against the rapeseed genome using the CLC main workbench 7.6.4 (CLC bio, Aarhus, Denmark). The target sequences are marked by arrows, the PAM sequence is marked by a blue line.

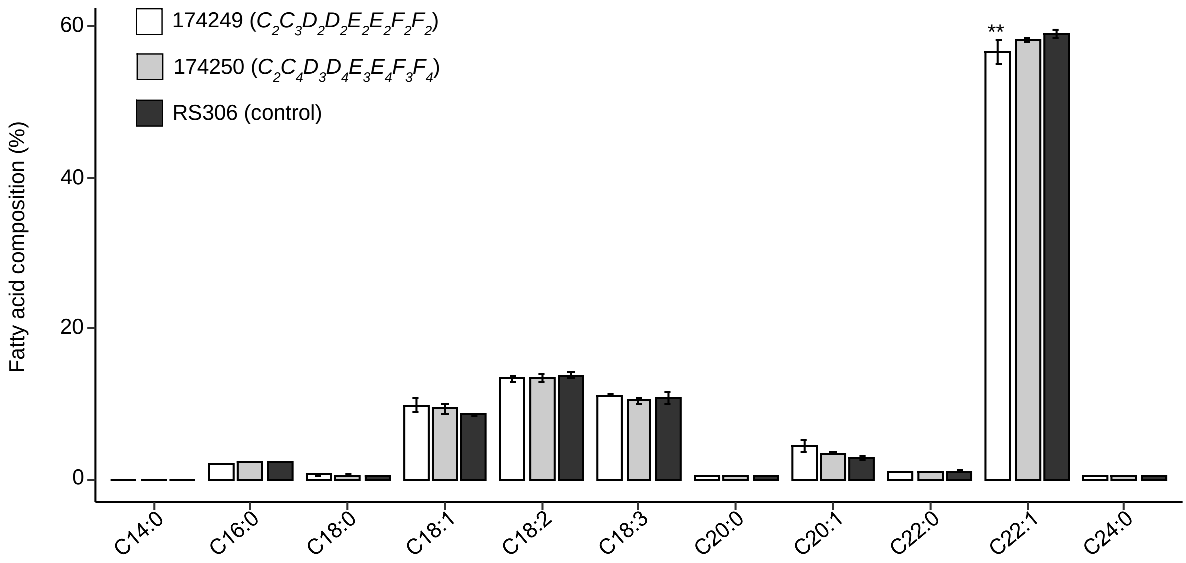

Figure S5: Fatty acid profiles in T_3 seeds of two *BnSFAR4* mutants and the RS306 control (n = 5). Fatty acid profiles were determined using gas chromatography. ***p* < 0.01 using a two-way ANOVA test, significant differences as compared to the RS306 controls. Data are presented as means ± SEM.

Supplementary Tables

Table S1: Features of the *BnSFAR* genes used in this study

Arabidopsis gene	*B. napus* sequence annotation	*B. napus* paralogs	Genomic sequence length (bp)	Exon/ Intron structure		Coding region (bp)	Protein size (aa)	Genome sequence identity with *AtSFAR* (%)	Amino acid sequence identity with *AtSFAR* (%)
				Exons	Introns				
AtSFAR1	*BnaC04g18090D*	*Bna.SFAR1.C04*	2310	5	4	1149	382	90.2	89.6
	BnaAnng12970D	*Bna.SFAR1.Ann*	2316	5	4	1149	382	83.0	90.1
AtSFAR2	*BnaA05g11260D*	*Bna.SFAR2.A05*	2006	4	3	1308	435	83.8	77.1
	BnaC04g13080D	*Bna.SFAR2.C04*	1254	3	2	1083	360	82.8	77.6
AtSFAR3	*BnaA05g34360D*	*Bna.SFAR3.A05*	1143	2	1	1053	350	86.5	90.6
	BnaC04g52870D	*Bna.SFAR3.C04*	1145	2	1	1053	350	84.9	93.5
AtSFAR4	*BnaA06g18900D*	*Bna.SFAR4.A06a*	1587	3	2	1149	382	85.6	84.8
	BnaC03g53090D	*Bna.SFAR4.C03a*	1460	3	2	1149	382	87.0	87.0
	BnaA06g16520D	*Bna.SFAR4.A06b*	2793	3	2	1149	382	85.6	82.3
	BnaCnng09560D	*Bna.SFAR4.Cnnb*	2803	3	2	1149	382	82.5	81.9
AtSFAR5	*BnaC07g35650D*	*Bna.SFAR5.C07*	1429	5	4	1083	360	88.8	90.7
	BnaA03g43830D	*Bna.SFAR5.A03*	1563	5	4	1086	361	88.5	90.9

Table S2: Primers used in this study. Red color indicates the mismatches introduced to increase the specificity according to Liu et al. (2012)[1]

B. napus paralog	Primer name	Primer sequence	Orientation	Primer combination	Purpose
Bna.SFAR1.C04	NK005	CAT GCT CTC TCG AAC AAA TTT CAA CCG	forward	NK005+NK032	RT-qPCR (Express 617)
	NK032	CTCCACCAATTCCACAACAGACCA	reverse		
	NK051	CAACCATATGGCTCCCACAGTAAACCAT	forward	NK051+NK052	TILLING
	NK052	ACGTGAGTGCAACACGTATGAAAGTT	reverse		
	NK131	AGATTTGAACCGAATAAATTGAGAGATGTA	reverse	NK051+NK131	Genotyping
	NK139	AGATTTGAACCGAATAAATTGAGAGATGTG	reverse	NK051+NK139	Genotyping
	HW001	TTCTTGCCTCTATTCCTTCCAC	forward	HW001+HW002	RT-qPCR (Hu135)
	HW002	GTTTGGCTGCTTGGTTATGG	reverse		
Bna.SFAR1.Ann	NK042	TGCCCTCTCGAACAAATTTCAAGCC	forward	NK042+NK012	RT-qPCR (Express 617)
	NK012	CCACCAGTTCCACAACAGAGCT	reverse		
	NK055	CAACCCTATGGCTCCCACTGTAAACCAG	forward	NK055+NK056	TILLING
	NK056	ACGTGAGTGCAACACATATGAAACCG	reverse		
	NK132	CCGGCTGCTGCAAAGTTACAACT	reverse	NK055+NK132	Genotyping

	NK140	CCGGCTGCTGCAAAGTTACAACC	reverse	NK055+NK140	Genotyping
	HW003	CAACACAGGACCGTTAGGATG	forward	HW003+HW004	RT-qPCR (Hu135)
	HW004	GTTTGGCTGCTTGGTTATGG	reverse		
Bna.SFAR4.A06a	NK033	GTGTCAAGCGACACTATTAGAGAA GTT	forward	NK033+NK023	RT-qPCR (Express 617)
	NK023	CATCTTCTGCAGCCAGCGACAAT	reverse		
	NK063	CAACCGCGG TCTCCGTCGC	forward	NK063+NK064	TILLING
	NK064	GTAGCCGACCCACACGTCTCGAAC	reverse		
	NK134	CGACTCACCATTGACTTCGTGGAGT	forward	NK134+NK023	Genotyping
	NK142	CGACTCACCATTGACTTCGTGGAGC	forward	NK142+NK023	Genotyping
	HW005	ACTGCCACGGATAGTTACGG	forward	HW005+HW006	RT-qPCR (Hu135)
	HW006	GGTTCGTTCCAAGTGTCTCC	reverse		
Bna.SFAR4.C03a	NK020	CTGTGTCAAGCGACACTATTAGAGGAT	forward	NK020+NK021	RT-qPCR (Express 617)
	NK021	CATCTTCTGCAGCCAGCGACTTC	reverse		
	NK060	CAACCGCCGTCTCCGTGGG	forward	NK060+NK062	TILLING
	NK062	GTTAATGTACCGACTCGGGTCCTTAGAC	reverse		
	NK136	GCGACTCACCATTGACTTCGTGGAGT	forward	NK136+NK021	Genotyping
	NK144	GCGACTCACCATTGACTTCGTGGAGC	forward	NK144+NK021	Genotyping
	HW007	ACTGCCACGGATAGTTACGG	forward	HW007+HW008	RT-qPCR (Hu135)
	HW008	GGTTCGTTCCAAGTGTCTCC	reverse		
Bna.SFAR4.A06b	NK037	CGC AAC CGC CGT CTC CAC	forward	NK037+NK038	RT-qPCR
	NK038	GTCGATGGTGAGTCGACCGTCAG	reverse		
	NK066	CAGGGATCTGATCTGCCATA TTCG	forward	NK066+NK068	TILLING
	NK068	CGACTGCTCGAGGTATCTTAAGCATGA**C**TA	reverse		
	HW009	CTCCCTTATCCTCGCAACC	forward	HW009+HW010	RT-qPCR (Hu135)
	HW010	CTGTGTCGGTGAATGAGTCG	reverse		
Bna.SFAR4.Cnnb	NK040	TCGCAACCGCCGTCTCCAT	forward	NK040+NK041	RT-qPCR (Express 617)
	NK041	CGATGGAGAGTCGACCGTCGC	reverse		
	NK072	GCATGCACTGTTGCTTGAAGAGCATATC	forward	NK072+NK073	TILLING
	NK073	CGACTGCTCGAGGTATCTTAAGCATGAGTT	reverse		
	NK138	CATAGTCATTTACTCCAATTTCTCCGAGCT	reverse	NK072+NK138	Genotyping
	NK146	CATAGTCATTTACTCCAATTTCTCCGAGCC	reverse	NK072+NK146	Genotyping
	HW011	ACGATGACTCTGGCTGATGA	forward	HW011+HW012	RT-qPCR (Hu135)
	HW012	GTGGCGTTCGGATACTTGAT	reverse		
Bna.SFAR5.A03	NK030	CAACGATCTCATCAACCGATACGCC	forward	NK030+NK031	RT-qPCR
	NK031	GCATTGATGTAAGTGAACTTAGCGTCTCCT	reverse		
	NK048	CTTCACGTTCTCAGACAAGTCTCTCTCCT	forward	NK048+NK049	Genotyping
	NK049	GTTGCTCGGTGTATCGGTTGATGAGA	reverse		

	NK107	AGAGTACTGGTTACCGGTGGAGTAGGAT	reverse	NK048+NK107	Genotyping
	HW013	TCGGTTTGGGAAGTAACGAC	forward	HW013+HW014	RT-qPCR (Hu135)
	HW014	CTCGGTGTATCGGTTGATGA	reverse		
Bna.SFAR5.C07	NK028	CAACGACCTTATCAACCGCTACGCT	forward	NK028+NK028	RT-qPCR (Express 617)
	NK029	GCATTGATGTAAGTGAACTTAGCATCTGCA	reverse		
	NK045	CTTCACGTTCTCAAAGCAGTCTCTCTCCA	forward	NK045+NK046	Genotyping
	NK046	GTTGCTCAGTGTAGCGGTTGATATGG	reverse		
	NK104	AGAGTACTGGTTCCCGGTGGAGTAATAG	reverse	NK045+NK107	Genotyping
	HW015	TGCCAACGACCTTATCAACC	forward	HW015+HW016	RT-qPCR (Hu135)
	HW016	GTCCCGTCTCTGCTGTTCTG	reverse		
Bna.SFAR2.A05	NK080	CTCCATCGTGTCCGAATCGCT	forward	NK080+NK081	RT-qPCR (Express 617)
	NK081	CCTTTACCTCTTGTTTTGCTACTGCAAG	reverse		
	HW017	GACCTAACCAGCCTCTCCACT	forward	HW017+HW018	RT-qPCR (Hu135)
	HW018	GCTTTCTTGTCTCCCACGAT	reverse		
Bna.SFAR2.C04	NK085	GCCATTCTAATCTTCGGCGACTCG	forward	NK085+NK088	RT-qPCR (Express 617)
	NK088	GACGAGCAATGTAATTCCTGAACATCGTA	reverse		
	HW019	CCTAACCAGCCTCTCCACTC	forward	HW019+HW020	RT-qPCR (Hu135)
	HW020	GCTTTCTTGTCTCCCACGAT	reverse		
Bna.SFAR3.A05	NK013	CCCGGCCTATCTTGATCCCTCC	forward	NK013+NK015	RT-qPCR (Express 617)
	NK015	GAAGTCGTTGGTTCCTATGCTGACTAG	reverse		
	HW021	CGTGTTGTGGAACTGGATTG	forward	HW021+HW022	RT-qPCR (Hu135)
	HW022	TTAGTCCTCTCGGTCGGATG	reverse		
Bna.SFAR3.C04	NK017	CCCGGCCTATCTGGATCCGTCT	forward	NK017+NK019	RT-qPCR (Express 617)
	NK019	GAAGTCGTTGGTTCCTATGCTGACCAA	reverse		
	HW023	AACGGGAGGTTGAGGAGATT	forward	HW023+HW024	RT-qPCR (Hu135)
	HW024	CACAACACGCAGAACTCGAT	reverse		
Bna.ACTIN2	Act-1	TCTGGTGATGGTGTGTCTCA	forward	Act-1+Act-2	RT-qPCR (Express 617)
	Act-2	GGTGAACATGTACCCTCTCTCG	reverse		
pCas9-TPC *bar* cassette	Cas1-f	CAGTCTTTCACCTCTCTTTGG	forward		Transgene verification
	Cas1-r	CCATCTTTGGGACCACTGTC	reverse		

Table S3: EMS-induced mutations in *BnSFAR1* and *BnSFAR4* genes. For each gene, one TILLING amplicon was used

	Bna.SFAR1.C04	***Bna.SFAR1.Ann***	***Bna.SFAR4.C03a***	***Bna.SFAR4.A06a***	***Bna.SFAR4.A06b***	***Bna.SFAR4.Cnnb***
Coding sequence coverage (%)	53.0	53.0	87.1	83.8	56.1	56.1
Number of M_2 pools used for mutation screening	4	8	8	10	10	4
Nonsense mutations	1	0	1	2	1	2
UTR mutations	0	0	0	0	1	0
Splice site mutations	1	1	2	1	1	0
Missense mutations	7	15	26	23	25	7
Silent mutations	2	4	15	12	8	5
Mutation frequency (1/kb)[a]	1/27.1	1/25.4	1/18.6	1/22.4	1/25.6	1/34.6

[a] Mutation frequencies were calculated as the number of mutations per M_1 plant based on the analyzed M_2 families.

Table S4: EMS and CRISPR-Cas mutations used for further studies

***B. napus* gene name**	**M_3/T_1 mutant code**	**Mutant type**	**Amino acid change**	**Allele**
**EMS induced mutations**				
Bna.SFAR1.C04	sfar1-1	C to T transitions	Glutamine to stop	A_1
Bna.SFAR1.Ann	sfar1-2	G to A transitions	Glycine to glutamic acid	B_1
Bna.SFAR1.Ann	sfar1-3	G to A transitions	Splice	B_2
Bna.SFAR4.A06a	sfar4-1	C to T transitions	Glutamine to stop	C_1
Bna.SFAR4.C03a	sfar4-2	C to T transitions	Glutamine to stop	D_1
Bna.SFAR4.A06b	sfar4-3	G to A transitions	Tryptophan to stop	E_1
Bna.SFAR4.Cnnb	sfar4-4	G to A transitions	Tryptophan to stop	F_1
Bna.SFAR1.C04	wild-type			A_E
Bna.SFAR1.Ann	wild-type			B_E
Bna.SFAR4.A06a	wild-type			C_E
Bna.SFAR4.C03a	wild-type			D_E
Bna.SFAR4.A06b	wild-type			E_E
Bna.SFAR4.Cnnb	wild-type			F_E
**CRISPR-Cas mediated mutations**				
Bna.SFAR4.A06a	bnsfar4-TP3	G insertion	Truncated protein (145/382 aa)	C_2
	bnsfar4-TP3	T insertion	Truncated protein (145/382 aa)	C_3
	bnsfar4-TP4	G insertion	Truncated protein (145/382 aa)	C_2
	bnsfar4-TP4	A insertion	Truncated protein (145/382 aa)	C_4
Bna.SFAR4.C03a	bnsfar4-TP3	T insertion	Truncated protein (145/382 aa)	D_2
	bnsfar4-TP4	A insertion	Truncated protein (145/382 aa)	D_3

	bnsfar4-TP4	1 bp deletion	Truncated protein (131/382 aa)	D_4
Bna.SFAR4.A06b	bnsfar4-TP3	G insertion	Truncated protein (145/382 aa)	E_2
	bnsfar4-TP4	T insertion	Truncated protein (145/382 aa)	E_3
	bnsfar4-TP4	A insertion	Truncated protein (145/382 aa)	E_4
Bna.SFAR4.Cnnb	bnsfar4-TP3	A insertion	Truncated protein (145/382 aa)	F_2
	bnsfar4-TP4	G insertion	Truncated protein (145/382 aa)	F_3
	bnsfar4-TP4	1 bp deletion	Truncated protein (131/382 aa)	F_4
Bna.SFAR5.A03	bnsfar5-TP1	21 bp deletion	Truncated protein (355/360 aa)	G_1
	bnsfar5-TP1	90 bp deletion	Truncated protein (94/360 aa)	G_2
	bnsfar5-TP1	143 bp deletion	deletion spanning intron<br>if only exon 2 lost, 320/ 360 aa	G_3
Bna.SFAR5.C07	bnsfar5-TP1	C insertion	Truncated protein (122/360 aa)	H_1
	bnsfar5-TP1	T insertion	Truncated protein (122/360 aa)	H_2
	bnsfar5-TP1	G insertion	Truncated protein (122/360 aa)	H_3
	bnsfar5-TP1	2 bp deletion	Truncated protein (121/360 aa)	H_4
	bnsfar5-TP1	4 bp deletion	Truncated protein (101/360 aa)	H_5
	bnsfar5-TP1	6 bp deletion	Truncated protein (358/360 aa)	H_6
	bnsfar5-TP1	28 bp deletion	Truncated protein (93/360 aa)	H_7
Bna.SFAR4.A06a	wild-type			C_R
Bna.SFAR4.C03a	wild-type			D_R
Bna.SFAR4.A06b	wild-type			E_R
Bna.SFAR4.Cnnb	wild-type			F_R
Bna.SFAR5.A03	wild-type			G_R
Bna.SFAR5.C07	wild-type			H_R

All EMS mutant alleles were combined to produce double mutants, except the B_2 mutant allele. The respective alleles from Express-617 (EMS donor) and RS306 (CRISPR-Cas donor) are named as suffix ‘E’ and ‘R’, respectively.

Table S5: Production of EMS mutants by crossing M_3 plants homozygous for the mutant allele

Crossing type	*B. napus* gene combination/ gene	M_3 Mutants	Amino acid change	F_2 Seed code	Genotype used for F_2 phenotyping
M_3 x M_3	*Bna.SFAR1.Ann* x *Bna.SFAR1.C04*	sfar1-1 x sfar1-2	Glutamine to stop x Glycine to glutamic	171769	$A_1A_1B_1B_1$, $A_1A_1B_EB_E$, $A_EA_EB_1B_1$, $A_EA_EB_EB_E$
M_3 x M_3	*Bna.SFAR4.A06a* x *Bna.SFAR4.C03a*	sfar4-1x sfar4-2	Glutamine to stop x Glutamine to stop	171770	$C_1C_1D_1D_1$, $C_1C_1D_ED_E$, $C_EC_ED_1D_1$, $C_EC_ED_ED_E$
M_3 x M_3	*Bna.SFAR4.A06b* x *Bna.SFAR4.Cnnb*	sfar4-3 x sfar4-4	Tryptophan to stop x Tryptophan to stop	171772	$E_1E_1F_1F_1$, $E_1E_1F_EF_E$, $E_EE_EF_1F_1$, $E_EC_ED_ED_E$
(M_3-Express) x (M_3-Express)	*Bna.SFAR1.Ann* x *Bna.SFAR1.C04*	sfar1-1 x sfar1-2	Glutamine to stop x Glycine to glutamic	180875	$A_1A_1B_1B_1$, $A_1A_1B_EB_E$, $A_EA_EB_1B_1$, $A_EA_EB_EB_E$
(M_3-Express) x (M_3-Express)	*Bna.SFAR4.A06a* x *Bna.SFAR4.C03a*	sfar4-1x sfar4-2	Glutamine to stop x Glutamine to stop	180876	$E_1E_1F_1F_1$, $E_1E_1F_EF_E$, $E_EE_EF_1F_1$, $E_EC_ED_ED_E$
(M_3-Express) x (M_3-Express)	*Bna.SFAR4.A06b* x *Bna.SFAR4.Cnnb*	sfar4-3 x sfar4-4	Tryptophan to stop x Tryptophan to stop	180877	$C_1C_1D_1D_1$, $C_1C_1D_ED_E$, $C_EC_ED_1D_1$, $C_EC_ED_ED_E$
M_3 x Express (F_2)	*Bna.SFAR4.A06a*	sfar4-1	Glutamine to stop	171780	C_1C_1, C_EC_E
M_3 x Express (F_2)	*Bna.SFAR4.C03a*	sfar4-2	Glutamine to stop	171782	D_1D_1, D_ED_E
M_3 x Express (F_2)	*Bna.SFAR4.A06b*	sfar4-3	Tryptophan to stop	171784	E_1E_1, E_EE_E
M_3 x Express (F_2)	*Bna.SFAR4.Cnnb*	sfar4-4	Tryptophan to stop	171786	F_1F_1, F_EF_E
F_1 x Express (BC_1F_2)	*Bna.SFAR4.A06a*	sfar4-1	Glutamine to stop	180886	C_1C_1, C_EC_E
F_1 x Express (BC_1F_2)	*Bna.SFAR4.C03a*	sfar4-2	Glutamine to stop	180887	D_1D_1, D_ED_E
F_1 x Express (BC_1F_2)	*Bna.SFAR4.A06b*	sfar4-3	Tryptophan to stop	180888	E_1E_1, E_EE_E
F_1 x Express (BC_1F_2)	*Bna.SFAR4.Cnnb*	sfar4-4	Tryptophan to stop	180889	F_1F_1, F_EF_E

Alleles from the non-mutated donor genotype Express 617 are named as suffix ‘E’

Table S6: Results of the *Agrobacterium*-mediated rapeseed hypocotyl transformation

Targeted ***B. napus*** paralogs	No. of hypocotyl explants	No. of regenerated shoots	No. of transgenic plants	No. of gene-edited plants
BnSFAR1	857	291	2	0
BnSFAR4	442	393	5	5
BnSFAR5	754	416	2	1

Table S7: Inheritance of CRISPR-Cas mutations in *BnSFAR4* and *BnSFAR5*. We analyzed T_2 offspring of four T_1 plants, bnsfar4-TP4, bnsfar4-TP3, bnsfar5-CP2 and bnsfar5-CP3 (seed codes below). O: observed number, E: expected number, H_R and G_R: wild type alleles from the donor genotype RS306. Allele names are given in Table S4.

Plant		Transgene		***sfar4* Genotypes**											
				Bna.SFAR4.A06a			***Bna.SFAR4.C03a***			***Bna.SFAR4.A06b***			***Bna.SFAR4.Cnnb***		
bnsfar4-TP4 (174250)		**Transgenic**	**Non-transgenic**	C_2C_2	C_2C_4	C_4C_4	D_3D_3	D_3D_4	D_4D_4	E_3E_3	E_3E_4	E_4E_4	E_3E_3	E_3E_4	E_4E_4
	O	11	7	4	7	7	4	12	2	5	7	6	6	11	1
	E	13.5	4.5	4.5	9	4.5	4.5	9	4.5	4.5	9	4.5	4.5	9	4.5
	χ^2	1.9[a]			1.9[b]			2.4[b]			1.0[b]			3.7[b]	
bnsfar4-TP3 (174249)				C_2C_2	C_2C_3	C_3C_3									
	O	9	1	2	4	4									
	E	7.5	3.5	2.25	4.5	2.25									
	χ^2	1.2[a]			1.2[b]										

Plant		Transgene		***sfar5* Genotypes**								
bnsfar5-CP2 (174257)		**Transgenic**	**Non-transgenic**	G_2G_2 H_1H_1	G_2G_2 H_1H_R	G_2G_2 H_RH_R	G_2G_R H_1H_1	G_2G_R H_1H_R	G_2G_R H_RH_R	G_RG_R H_1H_1	G_RG_R H_1H_R	G_RG_R H_RH_R
	O	36	0	5	6	2	2	8	1	4	4	4
	E	27	9	2.25	4.5	2.25	4.5	9	4.5	2.25	4.5	2.25
	χ^2	12.0[a]						10.89[c]				
bnsfar5-CP3 (174258)				G_RG_R H_5H_5	G_RG_R H_5H_R	G_RG_R H_RH_R						
	O	23	0	3	14	6						
	E	17.25	5.75	5.75	11.5	5.75						
	χ^2	7.7[a]			1.87[b]							

[a]3:1 segregation, χ^2 (df =1 at p = 0.05) = 3.84, [b]1:2:1 segregation, χ^2 (df =2 at p = 0.05) = 5.99, [c]1:2:2:1:4:1:2:2:1 segregation, χ^2 (df =8 at p = 0.05) = 15.5. Here we assume that T_1 parents were non-chimeric.

Table S8: Phenotyping data of EMS and CRISPR-Cas *BnSFAR1*, *BnSFAR4* and *BnSFAR5* mutants. T_2 and T_3 generations were derived from selfing of T_1 plants. F_1 plants are offspring from M_3 mutants crossed with the EMS donor Express 617. TKW: Thousand Kernel Weight, N: number of plants investigated. All data are means ± SEM.

**Mutant crossings**	**Generation**	**Seed code**	**Parental generation**	**Genotype**	**N**	**Seed oil content**	**TKW**
**EMS induced mutations**							
Bna.SFAR1.C04 x *Bna.SFAR1.Ann*	F_3	171769	M_3	$A_1A_1B_1B_1$	7	39.05±0.63	3.50±0.28
				$A_1A_1B_EB_E$	5	38.67±1.64	3.27±0.20
				$A_EA_EB_1B_1$	6	38.98±0.83	3.63±0.29
				$A_EA_EB_EB_E$	6	40.38±1.41	3.75±0.19
				Express 617	7	44.66±0.45	3.14±0.12
Bna.SFAR4.A06a x *Bna.SFAR4.C03a*	F_3	171770	M_3	$C_1C_1D_1D_1$	5	41.70±0.17	2.53±0.26
				$C_1C_1D_ED_E$	5	39.82±0.83	2.47±0.28
				$C_EC_ED_1D_1$	5	38.22±1.45	2.87±0.16
				$C_EC_ED_ED_E$	5	37.04±0.74	2.44±0.20
				Express 617	5	43.68±0.30	2.39±0.13
Bna.SFAR4.A06b x *Bna.SFAR4.Cnnb*	F_3	171772	M_3	$E_1E_1F_1F_1$	5	39.17±1.12	4.25±0.32
				$E_1E_1F_EF_E$	7	36.84±0.40	3.87±0.14
				$E_EE_EF_1F_1$	8	38.16±0.51	3.65±0.11
				$E_EC_ED_ED_E$	6	35.50±0.65	3.68±0.31
				Express 617	7	44.66±0.45	3.14±0.12
Bna.SFAR4.A06a	F_3	171780		C_1C_1	5	40.75±0.94	3.34±0.34
				C_EC_E	5	40.97±0.24	2.96±0.33
Bna.SFAR4.C03	F_3	171782		D_1D_1	5	41.02±1.01	3.08±0.14
				D_ED_E	5	40.54±0.94	3.33±0.22
Bna.SFAR4.A06b	F_3	171784		E_1E_1	6	42.16±0.86	3.22±0.20
				E_EE_E	6	41.34±0.40	3.64±0.20
Bna.SFAR4.Cnnb	F_3	171786		F_1F_1	5	41.00±0.60	3.62±0.30
				F_EF_E	5	39.04±0.12	3.13±0.12
Bna.SFAR1.C04 x *Bna.SFAR1.Ann*	F_3	180875	F_1(M_3-Express)	$A_1A_1B_1B_1$	5	41.25±0.59	4.06±0.34
				$A_1A_1B_EB_E$	6	41.51±0.51	4.15±0.25
				$A_EA_EB_1B_1$	5	40.23±1.21	3.81±0.24
				$A_EA_EB_EB_E$	7	41.50±0.75	3.65±0.22
Bna.SFAR4.A06a x *Bna.SFAR4.C03a*	F_3	180876	F_1(M_3-Express)	$E_1E_1F_1F_1$	6	44.96±0.31	3.92±0.12
				$E_1E_1F_EF_E$	6	44.23±0.35	3.72±0.28

				$E_{E}E_{E}F_{1}F_{1}$	5	44.24±0.57	3.62±0.29
				$E_{E}C_{E}D_{E}D_{E}$	5	41.28±0.65	3.47±0.28
Bna.SFAR4.A06b x *Bna.SFAR4.Cnnb*	F_3	180877	F_1(M_3-Express)	$C_{1}C_{1}D_{1}D_{1}$	7	42.33±0.69	4.67±0.25
				$C_{1}C_{1}D_{E}D_{E}$	5	40.18±0.74	4.12±0.32
				$C_{E}C_{E}D_{1}D_{1}$	6	40.11±0.65	3.96±0.20
				$C_{E}C_{E}D_{E}D_{E}$	6	38.91±0.79	3.71±0.32
				Express 617	8	45.50±0.48	3.78±0.09
Bna.SFAR4.A06a	F_3	180886		$C_{1}C_{1}$	5	44.82±0.90	4.55±0.25
				$C_{E}C_{E}$	5	43.42±1.29	4.00±0.44
Bna.SFAR4.C03	F_3	180887		$D_{1}D_{1}$	4	43.00±0.74	4.00±0.17
				$D_{E}D_{E}$	6	42.91±0.64	3.87±0.17
Bna.SFAR4.A06b	F_3	180888		$E_{1}E_{1}$	5	43.05±0.56	3.91±0.11
				$E_{E}E_{E}$	6	42.88±0.63	3.46±0.16
Bna.SFAR4.Cnnb	F_3	180889		$F_{1}F_{1}$	5	45.31±0.63	3.84±0.20
				$F_{E}F_{E}$	6	44.57±0.60	3.74±0.32
**CRISPR-Cas9 mediated mutations**							
BnSFAR4	T_2	174249		$C_{2}C_{3}D_{2}D_{2}E_{2}E_{2}F_{2}F_{2}$	5	36.21±0.30	6.25±0.12
BnSFAR4	T_2	174250		$C_{2}C_{4}D_{3}D_{4}E_{3}E_{4}F_{3}F_{4}$	5	34.66±0.92	5.56±0.36
				RS306	5	31.61±0.88	6.04±0.22
BnSFAR4	T_3	182975		$C_{3}C_{3}D_{2}D_{2}E_{2}E_{2}F_{2}F_{2}$	8	37.58±0.34	5.33±0.28
				RS306	8	33.29±0.49	4.48±0.27
BnSFAR5	T_2	174257		$G_{2}G_{2}H_{1}H_{1}$	4	34.44±0.87	6.15±0.82
				RS306	5	31.19±0.93	5.35±0.41
BnSFAR5	T_3	183033		$G_{2}G_{2}H_{1}H_{1}$	6	35.04±0.33	5.52±0.53
				RS306	7	31.66±0.35	5.45±0.38

Table S9: Seed germination, root, and shoot growth 5 DAS in T_3 lines with *BnSFAR* knock-out mutations and in RS306. All data are means ± SEM.

Seed code	Genotype	Germination rate (%)	Root growth (mm)	Shoot growth (mm)
174249	$C_2C_3D_2D_2E_2E_2F_2F_2$	97.2±2.6[a]	75.7±7.3[a]	59.0±6.4[a]
174250	$C_2C_4D_3D_4E_3E_4F_3F_4$	97.8±1.0[a]	76.5±5.8[a]	60.0±5.2[a]
RS306 (control)	$C_EC_ED_ED_EE_EE_EF_EF_E$	96.4±2.1[a]	76.0±13.0[a]	62.8±13.2[a]

Supplementary Dataset S1

1: SOC and fatty acid composition of 870 rapeseed accessions

2: SOC and fatty acid composition of all accessions for *SFAR1-SFAR5*

3: SOC and fatty acid composition of all accessions for *BnSFAR1*

4: SOC and fatty acid composition of all accessions for *BnSFAR2*

5: SOC and fatty acid composition of all accessions for *BnSFAR3*

6: SOC and fatty acid composition of all accessions for *BnSFAR4*

7: SOC and fatty acid composition of all accessions for *BnSFAR5*

Supplementary Dataset S2

1: *BnGDSL* genes expressed in developing seeds

2: *AtGDSL* orthologs equally expressed at 16 DAP and 40 DAP

3: *AtGDSL* orthologs upregulated at 40 DAP relative to at 16 DAP

4: *AtGDSL* orthologs downregulated at 40 DAP relative to at 16 DAP

References

1. Liu, J. et al. An improved allele-specific PCR primer design method for SNP marker analysis and its application. *Plant Methods* **8**, 34 (2012).
